# Heterogeneity of tumor microenvironment is associated with clinical prognosis of non-clear cell renal cell carcinoma: a single-cell genomics study

**DOI:** 10.1038/s41419-022-04501-9

**Published:** 2022-01-11

**Authors:** Wen-jin Chen, Hao Cao, Jian-wei Cao, Li Zuo, Fa-jun Qu, Da Xu, Hao Zhang, Hai-yi Gong, Jia-xin Chen, Jian-qing Ye, Si-shun Gan, Wang Zhou, Da-wei Zhu, Xiu-Wu Pan, Xin-gang Cui

**Affiliations:** 1 Department of Urology, The Third Affiliated Hospital of Second Military Medical University, 700 North Moyu Road, Shanghai, 201805 China; 2 School of Life Science and Biopharmaceutics, Shenyang Pharmaceutical University, Shenyang, 110016 China; 3 Peking-Tsinghua Center for Life Sciences, Tsinghua University, 100084 Beijing, China; 4 Department of Urology, Xinhua Hospital, Shanghai Jiaotong University, School of Medicine, 1665 Kongjiang Road, Shanghai, 200092 China; 5 Department of Urology, The Affiliated Changzhou No. 2 People’s Hospital of Nanjing Medical University, 29 Xinglong Road, Changzhou, 213000 Jiangsu China; 6 Department of Orthopedic Oncology, Changzheng Hospital of Second Military Medical University, 415 Fengyang Road, Shanghai, 200003 China

**Keywords:** Cancer genomics, Cancer microenvironment, Renal cell carcinoma

## Abstract

Non-clear renal cell carcinomas (nccRCCs) are less frequent in kidney cancer with histopathological heterogeneity. A better understanding of the tumor biology of nccRCC can provide more effective treatment paradigms for different subtypes. To reveal the heterogeneity of tumor microenvironment (TME) in nccRCC, we performed 10x sing-cell genomics on tumor and normal tissues from patients with papillary renal cell carcinoma (pRCC), chromophobe RCC (chrRCC), collecting duct carcinoma (CDRCC) and sarcomatoid RCC (sarRCC). 15 tissue samples were finally included. 34561 cells were identified as 16 major cell clusters with 34 cell subtypes. Our study presented the sing-cell landscape for four types of nccRCC, and demonstrated that CD8+ T cells exhaustion, tumor-associated macrophages (TAMs) and sarcomatoid process were the pivotal factors in immunosuppression of nccRCC tissues and were closely correlated with poor prognosis. Abnormal metabolic patterns were present in both cancer cells and tumor-infiltrating stromal cells, such as fibroblasts and endothelial cells. Combined with CIBERSORTx tool, the expression data of bulk RNA-seq from TCGA were labeled with cell types of our sing-cell data. Calculation of the relative abundance of cell types revealed that greater proportion of exhausted CD8+ T cells, TAMs and sarRCC derived cells were correlated with poor prognosis in the cohort of 274 nccRCC patients. To the best of our knowledge, this is the first study that provides a more comprehensive sight about the heterogeneity and tumor biology of nccRCC, which may potentially facilitate the development of more effective therapies for nccRCC.

## Introduction

The new diagnosis of kidney cancer is estimated to reach 73,750 cases in 2020 [1]. The proportion of renal cell carcinoma (RCC) in kidney cancer is up to 85% [2], of which clear cell RCC (ccRCC) and non-clear cell RCC (nccRCC) account for 75% and 25%, respectively, approximately [3]. Recent years have witnessed the critical progress in the management options of RCC to achieve better outcomes. Of those, the surgery remains the essential treatment and the targeted therapy involving VEGF, mTOR, or immunotherapy has also improved the patients’ survival [4, 5]. However, the pathogenesis and therapeutic targets of nccRCC are rarely elaborated and its therapeutic outcome remains unsatisfactory.

Papillary (pRCC, 10–15% of RCC), chromophobe (chrRCC, 5% of RCC), and collecting duct (cdRCC, aggressive, 1% of RCC) rank the three top subtypes of nccRCCs with controversial clinical understanding and therapy [6]. Sarcomatoid RCC (sarRCC, <1% of RCC) is also listed in the nccRCC classification of WHO with high mortality [6, 7]. Several small direct comparative clinical studies currently available have shown that the targeted therapies for nccRCC are not significantly different [8, 9], but it is difficult to determine the optimal treatment or reach consensus due to the limited data concerning the efficacy of the present therapies on nccRCCs [7]. Therefore, the development of drugs for efficient therapies against nccRCCs is still a great challenge.

Tumors are characterized by substantial heterogeneity, which can lead to different responses to the same therapy in patients. Hitherto, arduous efforts have been made to explain the heterogenetic features of tumors, but the understanding of tumor heterogeneity remains limited to tumor cells themselves [10]. Recent studies have demonstrated that the immune cells infiltrated by tumor and stromal cells present heterogeneity [11]. In addition, increasing evidence has shown that the tumor microenvironment (TME) plays an important role in the targeted drugs [12]. Previous studies have also emphasized that CD8+ exhaustion, immune checkpoints, tumor-associated macrophages (TAMs) [13] and cancer-associated fibroblasts (CAFs) [14] are critical therapeutic targets. All these data enhances our understanding about the heterogeneity of TME.

The concept of bulk RNA-seq is established on the assumption that each single gene is equally expressed in each cell, which could not reflect the true heterogeneity of tumor cells or TME. Thus, scRNA-seq is a major breakthrough to achieve single-cell transcriptome landscape, making it feasible to reveal the comprehensive TME or intratumoral heterogeneity. In the published scRNA-seq study of kidney cancer, Young et al. [15] described the single-cell profile for ccRCC, pRCC, and Wilms tumor. However, their study has not included the other nccRCCs, such as chrRCC and cdRCC. Thus, we further conducted the scRNA-seq for nccRCCs combined with the published pRCC samples to investigate the heterogeneity of nccRCCs and their TME, and explore potential direction for nccRCCs therapy. More importantly, we attempted to validate the clinical value of identified cell clusters through linking the scRNA data to the published datasets from TCGA nccRCC cohort (KIRP and KICH dataset), hoping that the result could help explain the features of nccRCCs and provide evidence-based data for developing novel clinical strategies for the treatment of nccRCC.

## Results

### Cell clustering of the nccRCC landscape

**Fig. 1:**
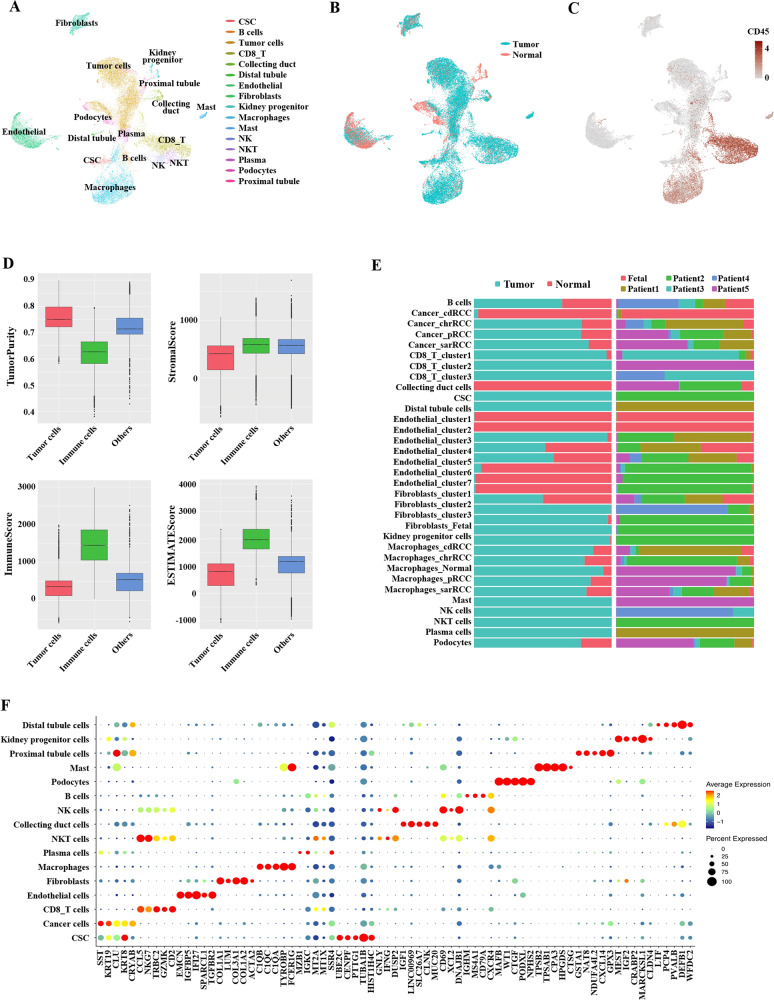
Landscape of single cells derived from nccRCC and normal tissues. **A** Sixteen major cell types identified by scRNA-seq; **B** sample origin (Tumor or normal); **C** immune cells (PTPRC+ or CD45+) or nonimmune cells; **D** the results of ESTIMATE algorithm: Tumor purity, Stromal score, Immune score of different cells; **E** for 34 subgroups identified in this landscape (left to right): the fraction of cells originating from 11 tumor and 4 normal samples, and the fraction of cells originating from each of 6 patients; **F** the bubble plot of top5 genes expression in each cell cluster; the size of bubble represents the percent expressed of cells; the color represents the average expression of each gene in clusters: red means the high expression.

A total of 14 tissues from five nccRCC patients were included in our work. Of the remaining cells after quality filtering, 27374 single cells were tumor-derived, and 7187 originated from non-malignant samples (Fig. 1E). These cells were classified into 16 major cell clusters: cancer cells and cancer stem cells (CSC), seven types of immune cells (PTPRC or CD45+), involving Mast, B cells, NK cells, NKT cells, Plasma cells, macrophages and CD8+ T cells, and nine nonimmune cell types (PTPRC or CD45−), involving distal tubule cells (DT), kidney progenitor cells, proximal tubule cells (PT), podocytes, collecting duct cells (CD), fibroblasts and endothelial cells (Fig. 1A–C). As described in Fig. 1D, all cells were classified into Tumor_cells, Immunue_cells and Others, and showed Tumor_cells had higher tumor purity, and Immune_cells had higher immune scores, confirming the relative accuracy of the clustering. Additionally, five top markers of each major cell type are shown in the form of a bubble diagram in Fig. 1F.

### nccRCC cancer cells present diversities and abnormality in metabolism and gene expression compared with corresponding normal-derived cells

**Fig. 2:**
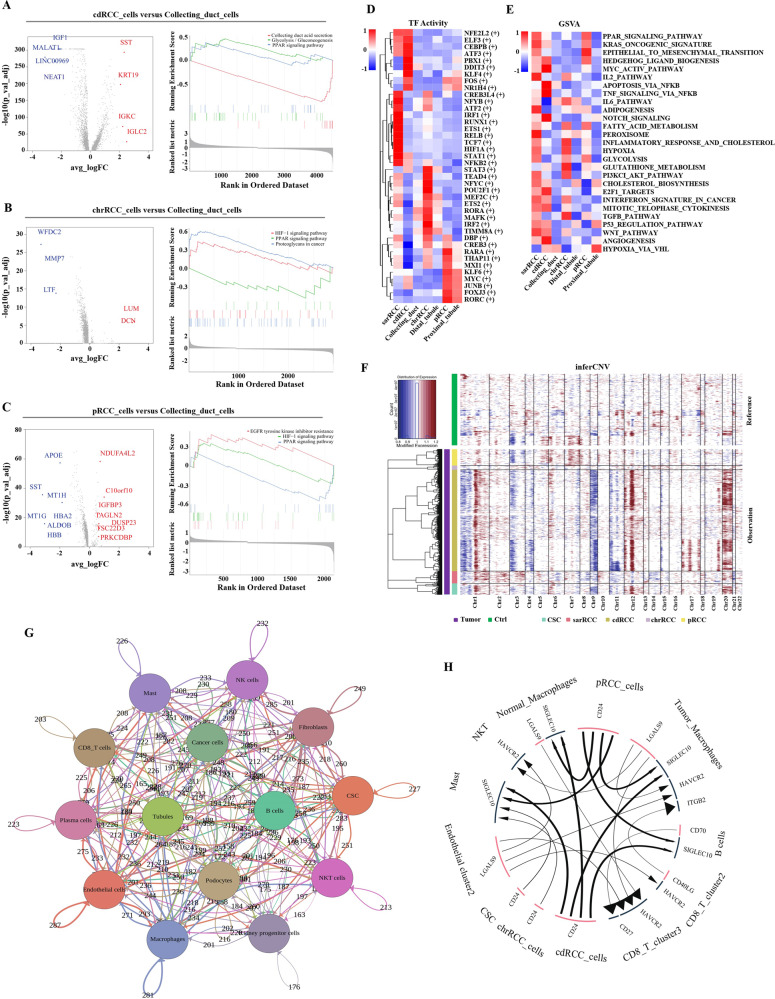
Abnormal biological processes occur in nccRCC. **A**–**C** Volcano plot of differentially expressed genes (DEGs) between cancer cells and corresponding normal-derived renal tubular epithelial cells. Upregulated genes are colored in red, and downregulated genes are colored in blue; and GSEA results revealed the pathways enriched in cancer cells; **D** Heatmap of selected regulons altered in cancer cells. AUC scores were measured by SCENIC per cell; **E** Heatmap of GSVA results for selected pathways altered in cancer cells; upregulated pathways are colored in red, and downregulated pathways are colored in blue; **F** InferCNV results of cancer cells, referenced by the normal-derived renal tubular epithelial cells. The sarRCC possessed amplifications from chromosomal 7 and deletions from chromosomal 8; the CDRCC possessed deletions from chromosomal 11, while the amplifications located on chromosomal 12; the CNVs of chrRCC occurred in chromosomal 4, 5, 17, 18. In pRCC, the loss was derived from chromosomal 3, while the amplifications occurred on chromosomal 22. **G** iTALK results revealed the interactions between the cell clusters. Immune checkpoints interactions of cancer cells with CD8+ T cells, macrophages and endothelial cells. Chemokines, including CCL5, CXCL12, CXCR3 and CXCR4, secreted by CD8+ T cells, macrophages and endothelial cells in TME were frequently interacted with nccRCC cells. VEGFA, PGF and IGF secreted by nccRCC cells actively interacted with CD8+ T cells, macrophages, endothelial cells and fibroblasts, which could be associated with angiogenesis. Each circle represents a cluster; the arrows represent the interactions between circles; the number on the arrows represent the number of the interactions; **H** The interaction of top 20 ligand-receptor pairs are presented. The size of the arrow represent the relative expression level of the receptor (thick for high, small for low); the size of the line represent the relative expression level of the ligand (thick for high, small for low).

CDRCC, chrRCC, pRCC originated from CD [16], DT [17], and PT [18], respectively. DEGs were analyzed respectively between CDRCC vs. CD, chrRCC vs. DT, pRCC vs. PT. The DEGs analysis of scRNA-seq was based on cell markers [19], so that the results could be regarded as the exclusive DEGs between cancer cells and corresponding normal-derived cells. Interestingly, we found that the upregulated or downregulated DEGs were potentially associated with the metabolic pathways (Figs. 2A–C and S1). Then, we conducted KEGG enrichment analysis of the altered genes. Previous study has reported that PPAR pathway was suppressed in ccRCC cells, compared with renal tubule cells [19]. However, our Gene Set Enrichment Analysis (GSEA) results showed that only pRCC cells presented the similar repressed PPAR pathway, which could be the result that pRCC and ccRCC both originate from PT [20]. Meanwhile, Glycolysis pathway was enriched for CDRCC, while the CD acid secretion pathway was repressed; the proteoglycan pathway in cancer was enriched in chrRCC (Fig. 2A–C).

Compared with the other nccRCC or normal tubules, HIF-1α was upregulated in sarRCC cells, which was previously demonstrated to be an independent prognostic factor in sarRCC [21]. Genes regulated by other TFs associated with lipid metabolism, involving KLF4, KLF6, CEBPB [22–24], were upregulated in nccRCC cells (Fig. 2D). As for autophagy related TFs, pRCC presented the similar results to ccRCC that NR1H4 was downregulated compared with PT [19]. On the contrary, NR1H4 was upregulated in CDRCC cells. Other ccRCC-associated TFs such as NFκB, STAT3, POU2F2 and RARA were also upregulated in nccRCC [19, 25]. The gene set variation analysis (GSVA) analysis also demonstrated that PPAR pathway was upregulated in PT (Fig. 2E). The hypoxia pathway was upregulated in all the nccRCC cells except CDRCC, which presented the active adipogenesis. The classical pathways, P53, EMT and angiogenesis, were upregulated in nccRCC cells [26–28]. And tumor glycolysis, glutathione metabolism pathways were upregulated in nccRCC cells [29, 30]. Both the SCENIC and GSVA analyses revealed that the NFκB pathway was upregulated in cancer cells. NFκB was involved in T cell exhaustion [31] and suppression of macrophages surveillance in tumor development [32]. Highly heterogenetic copy number variations (CNVs) were obtained from these types of nccRCCs (Fig. 2F), whose results were close to the previous study [33]. Finally, the active interactions occurred between cancer cells and CD8+ T cells, macrophages, endothelial cells and fibroblasts; the top 20 ligand-receptor pairs and the top 20 interactions of immune checkpoints, cytokines, growth factors in TME were shown (Fig. 2G, H).

### Exhausted CD8+ T cells tend to progress in TME of nccRCC

**Fig. 3:**
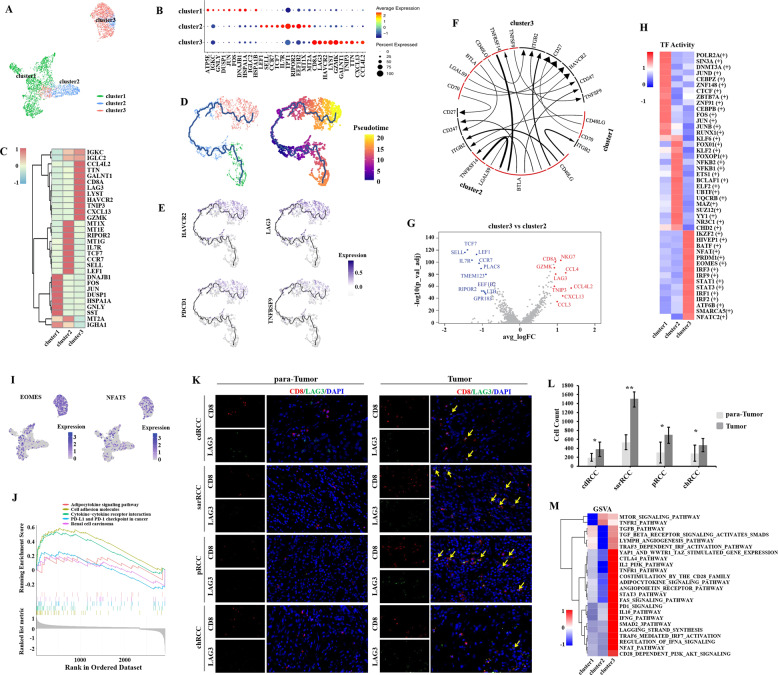
Exhausted CD8+ T cells are enriched in nccRCC TME. **A** UMAP plot of three subsets of CD8+ T cells; **B**, **C** Dotplot of five top markers of each cell cluster; diameters of dots represent abundance, and color represents the expression level; **D** Differentiation trajectory of CD8+ T cells in nccRCC, with each color coded for pseudotime (left) and clusters (right); **E** Distribution of immune checkpoint expression in CD8+ T cells; **F** iTALK analysis revealed the interaction of immune checkpoints among the three clusters; the size of the arrow represent the relative expression level of the receptor (thick for high, small for low); the size of the line represent the relative expression level of the ligand (thick for high, small for low). **G** Volcano plot of upregulated and downregulated genes between clusters 3 and 2 (logFC > 0.9, *p* < 1e−5); upregulated genes are colored in red, and downregulated genes are colored in blue. **H** Heatmap of expression regulated by transcription factors estimated by SCENIC among the three clusters; upregulated TFs are colored in red, and downregulated TFs are colored in blue; **I** The distribution of EOMES and NFAT5 expression in CD8+ T cells, which participates in T cell dysfunction; **J** GSEA revealed pathways enriched in exhausted T cells. FDR < 0.05 was considered as significantly enriched. **K**, **L** The IHC analysis results of CD8+LAG3+ cells between para-Tumor and Tumor tissues. (random 20 fields and bar = 50 μm). **M** GSVA results of pathways enriched in exhausted T cells, and most cancer-associated pathways upregulated in exhausted T cells. Upregulated pathways are colored in red, and downregulated pathways are colored in blue.

A total of 3701 CD8+ T cells were detected and then divided into three sub-clusters (Fig. 3A). The top 10 markers are presented in the bubble chart (Fig. 3B). The immune checkpoints were detected, involving LAG3, HAVCR2, PDCD1, CTLA4, TNFRSF9 (Fig. 3C). Notably, the immune checkpoint markers were upregulated in cluster 3 (Fig. 3E), and thus we confirmed that cluster 3 were exhausted in nccRCC TME. Currently, PD-1/PD-L1 and CTLA4 are popular for immunotherapy targets, even though fewer than 50% patients with solid tumors benefited from this treatment. However, there were no prominent evidence demonstrating that immune checkpoints targeted immunotherapy could be helpful for nccRCC. Our data may provide an understanding that the HAVCR2/LAG3 could be the better target for the exhausted CD8+ T cell subpopulation of nccRCC, and they might be even superior to PD-1/PD-L1 and CTLA4 as the immunotherapy target for nccRCC.

Previous studies have demonstrated the role of CD8+ T cell exhaustion in RCC [34]. We used Monocle 3 to perform the differentiation of trajectory, visualized in Fig. 3D. Non-malignant tissues derived CD8+ T cells (cluster 1) could differentiate into cluster 2 and cluster 3. The cluster 3, representing exhausted CD8+ T cells, presented at the end direction. Cluster 3 expressed more immune checkpoints related ligand-receptor pairs, while cluster 2 possessed more normal ligand-receptor pairs CD8+ T cells with immunity. DEGs between cluster 3 vs. 2 were presented in the volcano plot (Fig. 3G).

The changes of TFs during the T cell exhaustion in nccRCC were displayed in Fig. 3H, I. These data suggest that NFAT might lead to the upregulation of genes associated with T cell dysfunction without FOS and JUN [35]. EOMES, the TF participating in the regulation of the end differentiation of T cells, was upregulated in cluster 3, with immunosuppressive NFAT5 (Fig. 3I). In addition, STAT3 was also observed to be upregulated (Fig. 3H). The GSEA analysis results were shown in Fig. 3J. IHC analysis for CD8+LAG3+ cells between para-Tumor and Tumor suggested that CD8+LAG3+ T cells potentially acted as the key exhaustion T cell marker in nccRCC (Fig. 3K, L, all *p* < 0.05). The GSVA results also showed that NFAT, STAT3, CTLA4, and PD1, along with other tumor potentially activated pathways were upregulated in the cells from cluster 3 (Fig. 3M).

### Macrophages play an important role and present tumor infiltration in nccRCC

**Fig. 4:**
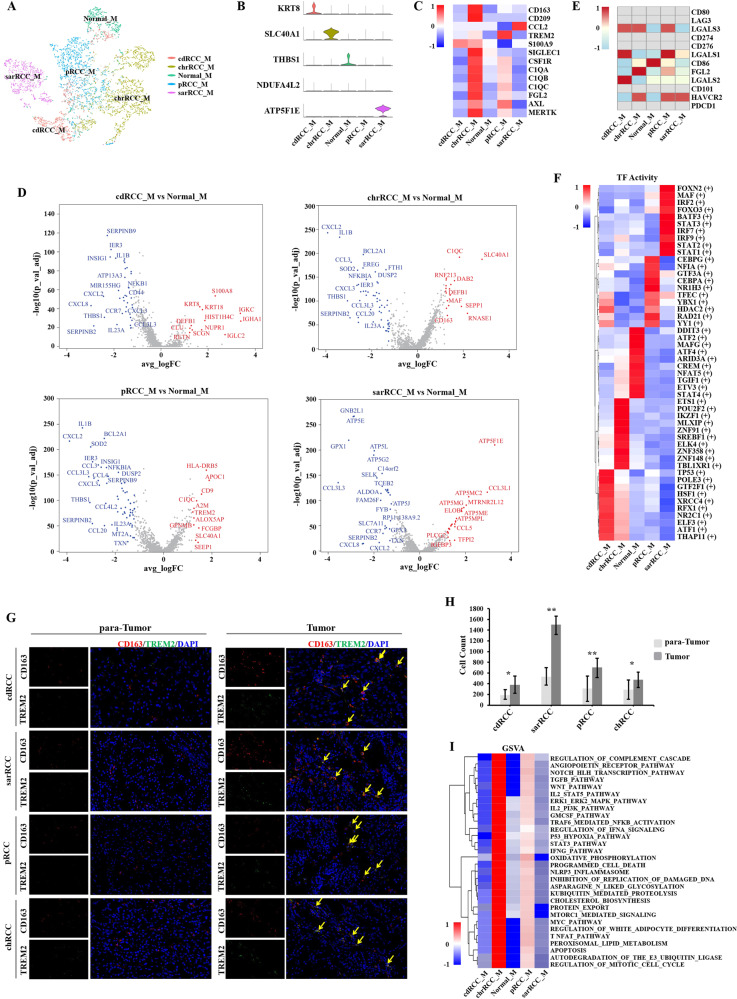
Tumor-infiltrating macrophages presented immunosuppressive in nccRCC TME. **A** tSNE plot of five subgroups of macrophages; **B** Violin plot of specific markers of each subgroup; **C** Heatmap of published marker of M2-poloarized like TAMs. Expression of each cluster was normalized into a row *Z* score; **D** Volcano plot of upregulated and downregulated genes between differently derived macrophages and normal macrophages; upregulated genes are colored in red, and downregulated genes are colored in blue. **E** Heatmap of already known immune checkpoints expression on macrophages. The row *Z* score represented the expression level; **F** Heatmap of expression regulated by transcription factors estimated by SCENIC among five clusters; red color represents active TFs; **G**, **H** The IHC analysis results of CD8+LAG3+ cells between para-tumor and tumor tissues. (random 20 fields and bar = 50 μm). **I** GSVA results of pathways enriched in each cluster of macrophages, and most cancer-associated pathways upregulated in tumor-infiltrating macrophages.

Of these 16 cell clusters, macrophages possessed the largest cell number (6404). As described in the Fig. 2G, H, macrophages interacted with nccRCC cells actively. This main cluster was sub-grouped into five cluster based on derivation (Fig. 4A). Next, we identified the markers of each subgroup, confirming that one cluster was derived from non-malignant tissues (Normal_M cluster, THBS1+), while pRCC_M (NDUFA4L2), chrRCC_M (SLC40A1), cdRCC_M (KRT8+) and sarRCC_M (ATP5F1E) clusters were enriched in corresponding nccRCC tissues (Fig. 4B). THBS1 has been reported to possess antiangiogenic and antitumor effects [36]; NDUFA4L2 may be the molecular target for ccRCC therapy [37]; SLC40A1 was found to play an inflammatory role in TAMs [38]; it was reported that the KRT8+ state could establish specific intercellular communication with mesenchyme and macrophages during injury repair [39].

The expression of TAMs markers was visualized in Fig. 4C, such as TREM2+. Subsequently, we calculated the DEGs for nccRCC macrophages (Fig. 4D). As described in Fig. 4E, the HAVCR2 was highly expressed in chrRCC, pRCC and sarRCC derived macrophages. Interestingly, the most popular immune checkpoints, PD1 and CTLA4 were not found to be different. Thus, HAVCR2 was more likely to be the therapy target for nccRCC rather than PD1 or CTLA4. In addition, FGL2, the immunosuppressive ligand for FcγRIIB on CD8+ T cells, inducing the apoptosis of effector CD8+ T cells [40], was highly expressed in chrRCC cells and relatively highly expressed in pRCC cells. Cell crosstalk between different clusters were in Fig. S2A. As shown in Fig. S2B, tumor-derived macrophages were at the end of differentiation trajectory.

SCENIC analysis (Fig. 4F) revealed that MAFG, a type of macrophage-activation factor promoting inflammation [41], was upregulated in the Normal_M. In addition, ATF2 and ATF4, the known component of activator-protein-1, were also upregulated in Normal_M, which could participate in inflammatory stimuli [42]. IRF2, upregulated in chrRCC_M, has been reported to be immunosuppressive, and IRF/STAT1 was also related to M2 polarization [43, 44]. The IHC analysis indicated that higher in tumor tissues expressed higher CD163+TREM2+ compared with para-tumor did (Fig. 4G, H). The tumor-associated pathways were mostly upregulated in chrRCC_M (Fig. 4I), which were close to the GSVA results of ccRCC [19].

### Fibroblasts present heterogeneity in nccRCC and endothelial cells exhibit diversities because of derivation

**Fig. 5:**
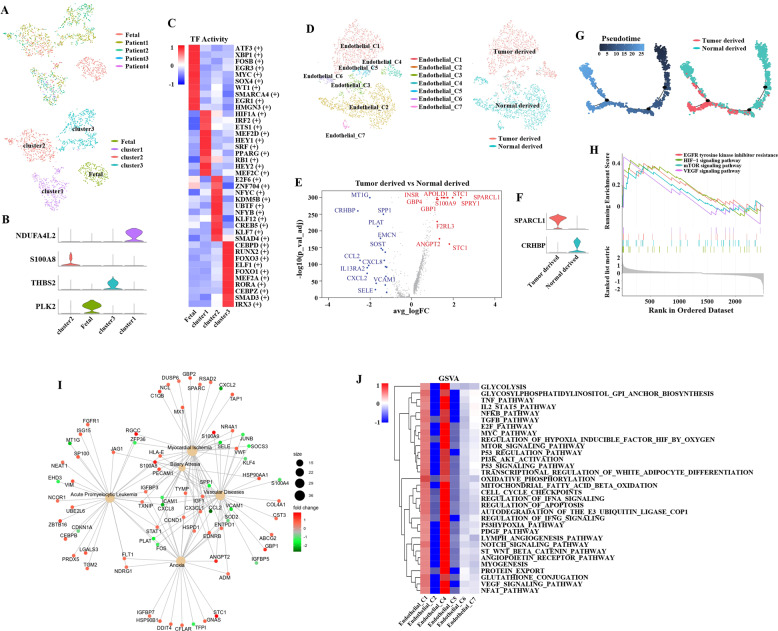
The characteristics of fibroblasts and endothelial cells in nccRCC TME. **A** tSNE plot of four subsets of fibroblasts; **B** Violin plot of specific markers of each subgroup in fibroblasts; **C** Heatmap of expression regulated by transcription factors estimated by SCENIC among the three clusters; red color represents active TFs. **D** tSNE plot of seven subsets of Endothelial cells; and the distribution of tumor-derived and normal-derived endothelials; **E** Volcano plot of upregulated and downregulated genes between tumor-derived and normal-derived macrophages (logFC > 1.15, *p* < 1e−5); upregulated genes are colored in red, and downregulated genes are colored in blue. **F** Violin plot of specific markers of tumor-derived and normal-derived macrophages; **G** Differentiation trajectory of macrophages in nccRCC, with each color coded for pseudotime (top) and clusters (bottom); **H** GSEA revealed pathways enriched in tumor-derived macrophages, FDR < 0.05 was considered as significantly enriched. **I** The pathways network of tumor-derived macrophages. **J** GSVA results of pathways enriched in each cell cluster, and most cancer-associated pathways upregulated in tumor-derived macrophages (C1 and C4).

A total of 2217 cells were identified as fibroblasts, and mostly were tumor-derived (Figs. 5A and S3): fetal cluster (PLK2), cluster 1 (NDUFA4L2), cluster 2 (S100A8), and cluster 3 (THBS2) (Fig. 5A, B). PLK2, a member of the polo-like kinase family, was differentially expressed after activation of quiescent fibroblasts [45]. NDUFA4L2 could participate in the oxidative phosphorylation of CAFs [46] and protect the cancer cells in low-oxygen consumption [47, 48]. In addition, IL-6 and IL-8 released by fibroblasts could stimulate the upregulation of S100A8 in tumor-infiltrating myeloid cells, and fibroblasts could promote the differentiation of cells in bone marrow into S100A8-expressed TAMs in TME [49]. Proteomic analysis showed that thrombospondin-B2 (THBS2) was correlated with angiogenesis when CAFs were activated in TME [50]. SCENIC analysis revealed that CEBPD, FOXO3, FOXO1, CEBPZ, and SMAD3 in the cluster 3 were upregulated (Fig. 5C). CEBP family was known to play a critical role in adipocyte differentiation [51]. FOXO1/3 and SMAD3 pathways could promote the tumor development [52]. Similarly, cells from cluster 1, HIF1A and PPARγ were upregulated and associated with lipid accumulation [53]. As shown in a recent 10x Genomics scRNA-seq study of ccRCC [19], CAFs in TME of nccRCC presented were associated with abnormal lipid metabolism. Meanwhile, the cell crosstalk between different fibroblasts clusters were presented in Fig. S2C. The differentiation of trajectory was visualized in Fig. S2D. The non-malignant tissues derived fibroblasts could differentiate into clusters 1–3, which could be all regarded as CAFs, as these clusters were tumor-derived (Fig. 5A).

As shown in the Fig. 5D, 5355 endothelial cells derived from tumor or normal tissues were clustered into seven subgroups. The DEGs are listed in the volcano plot (Fig. 5E). The SPARCL1+ and CRHBP+ were identified for tumor and normal derivation respectively (Fig. 5F). SPARCL1, a matricellular protein, can increase the neo-angiogenetic and infiltrative features of tumor [54] and regulate the TME-dependent heterogeneity of endothelial cells [55]. CRHBP, as the CRH binding protein, could upregulate NF-κB and p53 induced apoptosis to suppress ccRCC cells development [56], here was characteristically detected in normal-derived tissues.

As shown in Fig. 5G, tumor-derived endothelial cells were at the end of differentiation trajectory, which was hypothesized that normal-derived endothelial cells, tended to evolve into tumor-associated ones. GSEA analysis revealed that RCC-associated pathways, like EGFR, HIF-1, mTOR, and VEGF, were upregulated in the tumor-derived endothelial cells, indicating the vascular or anoxia-related diseases occurred in the endothelial cells of TME in nccRCC (Fig. 5H, I) and the tumor-educated process could be the angiogenesis-induced alternations. Subsequently, GSVA results revealed the heterogeneity in different derived endothelial cells (Fig. 5J). Clusters 1 and 4 were both tumor-derived and WNT-b-catenin, NOTCH signaling, angio-protein, glycolysis, hypoxia pathways were upregulated and enriched in these cells.

### sarRCC cells or infiltrative immune cells in TME are correlated with the poor outcomes in an acknowledged 274 patients nccRCC cohort

**Fig. 6:**
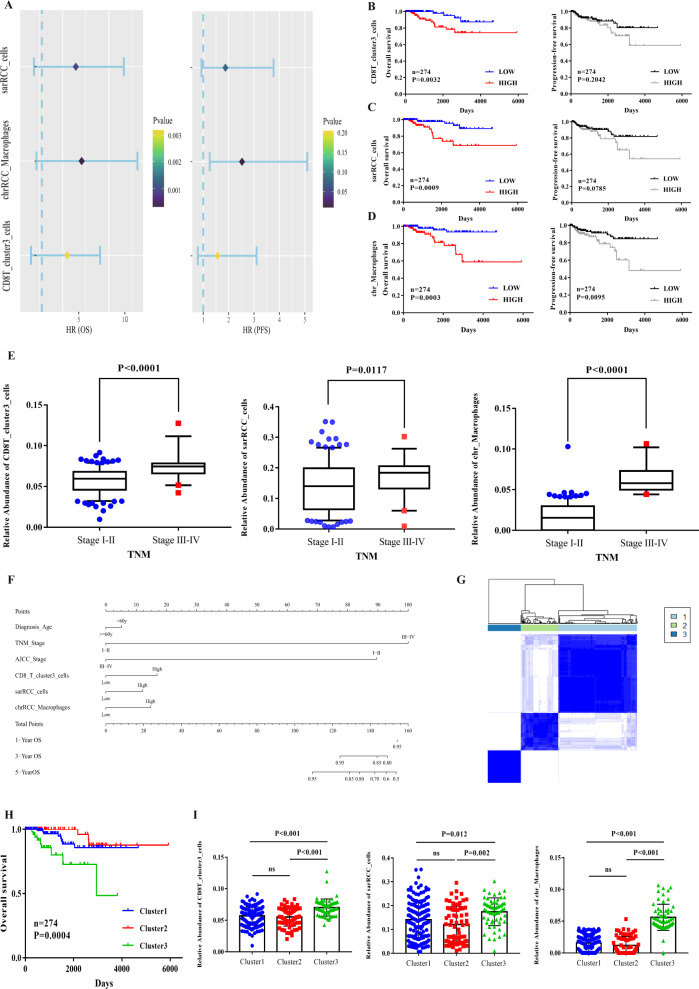
Clinical value of cell clusters of TME recognized by our scRNA-seq analysis in patients of TCGA nccRCC cohort. **A** Forest plot of cell clusters calculated by CIBERSORTx and overall survival (left) and progression-free survival (right); hazard ratios (HRs), with their 95% confidence intervals. **B**–**D** Kaplan–Meier survival curve for patients in TCGA nccRCC. Log rank *p* value <0.05 was considered as statistically significant; **E** Boxplots showed that CD8T_cluster3_cells, sarRCC_cells and chrRCC-derived macrophages were enriched in high-TNM stage; **F** Nomogram plot presented the prognostic role of significant cell clusters combined with clinical factors; **G** nccRCC patients from TCGA nccRCC were divided into three clusters by ConsensusClusterPlus according to cell clusters recognized in this landscape; **H** Survival analysis of three patient clusters. Log rank *p* value was calculated in GraphPad Prism 7.0; **I** Relative abundance of CD8T_cluster3_cells, sarRCC_cells and chrRCC-derived macrophages in three patient clusters. Tukey’s multiple comparison test was used.

Finally, we calculated the relative abundance of each cluster in patients of TCGA nccRCC cohort by CIBERSORTx to evaluate the clinical significance of these cell clusters. As visualized in Fig. 6A, B, C, D, the cells from CD8T_cluster3_cells (Exhausted T cells), chrRCC_macrophages, and sarRCC_cells were all correlated with worse OS and PFS (HR > 1 and *p* < 0.05). Interestingly, the abundance of these three cell clusters was increased in the high-TNM-stage patients (Fig. 6E, sequentially *p* < 0.0001, *p* = 0.0117, *p* < 0.0001). Subsequently, we established a clinical predictive model to evaluate the correlation of abundance of the three cell clusters with age, TNM stage and AJCC stage, and found that the multi-factors may favorably affect the predictive ability (Fig. 6F). To further explore the clinical significance of immune TME, we categorized the 274 nccRCC patients into three immune groups via ConsensusClusterPlus (“Materials and methods”, Fig. 6G). Compared with the patients of Clusters 1 and 2, those from Cluster 3 presented worse OS (Fig. 6H, *p* = 0.0004). Notably, we found that the relative abundances of CD8T_cluster3_cells (Exhausted T cells), chrRCC_macrophages and sarRCC_cells in Cluster 3 of ConsensusClusterPlus were all significantly higher than those in the other two clusters (Fig. 6I, sequentially *p* < 0.0001, *p* = 0.012, *p* < 0.0001). Therefore, these data pointed out the critical significance of exhausted CD8+ T cells, TAMs and sarcomatid RCC in nccRCC progression.

## Discussion

Besides ccRCC, nccRCCs also make the treatment of renal tumors an arduous task, especially due to the high malignancy of some subtypes. Due to no available standardized systemic diagnosis or management options for nccRCC, conventional renal cancer treatments such as mTOR, TKIs, chemotherapy and immune checkpoint inhibitors remain the main therapies [57]. However, the efficacy of these treatments is limited. It has been gradually recognized that tumor-infiltrating cells can affect the response to treatments or aggravate the drug resistance in TME [5, 58], and therefore more attention should be paid to not only nccRCC cells but TME cells for the sake acquiring optimal therapeutic approaches. In this work, we performed scRNA-seq to investigate nccRCC and described a complete profile of human nccRCC tissues in TME. Moreover, we recognized the new cell subsets and changed pathways in nccRCC, as well as TFs driven regulatory networks. Our data will contribute to the understanding of TME heterogeneity of nccRCC and explore new targets for nccRCC treatments.

Due to the different tumor subtypes and high complexity of TME in nccRCCs, their intratumoral heterogeneity cannot be fully described. However, several critical observations should be noted. Firstly, abnormal biological characteristics, like lipid accumulation, have been reported before, but the underlying mechanisms are still ambiguous. Previous ccRCC study rarely observed that PPAR pathway dominated in the process of lipid accumulation [19]. However, we noticed that PPAR pathway was downregulated only in pRCC, which was different from ccRCC. The reason could be that previous studies failed to find that ccRCC originates from PT, so that they simply compare ccRCC and all tubules cells. In cdRCC, we unveiled the important role of glycolysis/gluconeogenesis pathway in the lipid metabolism. Meanwhile, we noticed that HIF-1 pathway was upregulated in both chrRCC and pRCC, which was also correlated with glycolysis and reprogram metabolism [59]. Moreover, each subtype of nccRCCs possessed several genes, which could be promising biomarkers and have significant clinical value for nccRCCs. Interestingly, the abnormality of metabolic biology occurred in both tumor cells and tumor-infiltrating cells or stromal cells. Thus, further studies on nccRCCs should pay attention to both cancer cells and other associated/infiltrating cells.

Secondly, we discovered that CD8+ T cells tended to be exhausted in nccRCC, which was correlated with poor prognosis. This might explain why CD8+ T cell infiltration was accompanied with a worse clinical outcome in the kidney cancer study [60]. We acquired the first pseudotime trajectory of CD8+ T cell exhaustion in nccRCC and revealed the pathways that were activated in several stages of the biological process. Novel strategies against nccRCC could emerge when these pathways are inhibited to recover the activated CD8+ T cells. Moreover, we noted some important TFs changes, which may potentially participate in T cell dysfunction. Our findings may provide useful clues for a better understanding about T cell dysfunction process in nccRCCs.

Thirdly, differently derived macrophages presented diverse phenotypes. Normal macrophages played an antitumor role, while different tumor tissues infiltrating macrophages showed cancer-associated pathways and TFs. More importantly, the subgroup of chrRCC infiltrating macrophages was correlated with poor prognosis in the cohort of TCGA nccRCC patients. Previous study reported that the conventional TAMs biomarker, CD68+CD163+ could not be fully adequate in ccRCC and the TREM2+ should be one marker to identify TAMs [19]. When compared with normal macrophages, TREM2+ was upregulated in all these nccRCC subtypes, which was consistent with previous study. In addition, our pathway, checkpoint and TFs analysis demonstrated that TAMs serve as an immunosuppressive part in the nccRCC TME. Our results will be helpful to explore and validate the novel targets for nccRCC immunotherapy.

Fourthly, the identification of nccRCC subtypes has usually been based on bulk RNA-seq or microarray analysis [61]. The 274 patients from TCGA nccRCC cohort were clustered into three different groups according to the proportion of cell subsets defined in our study for the first time. Remarkably, patients in consensus cluster 3 presented poorer clinical prognosis. The significance of T cell exhaustion in nccRCC was further confirmed by the fact that the relative abundance of exhausted CD8+ T cells was low in clusters 1 and 2, and was enriched in cluster 3, though larger prospective cohort studies are needed to further investigate and validate our findings. In addition, although this scRNA-seq analysis performed in-depth characterization of nccRCC and its TME in molecular levels, limitations for multiple samples from the six patients might still exist. As nccRCC is relatively rare, more nccRCC fresh samples are difficult to obtain. On one hand, different lesions of RCC also present heterogeneity in previous work [62]. Thus, our samples from primary tumor and metastatic sites served as a basis for exploring the TME and heterogeneity of nccRCC. It was the first time to display the relative comprehensive nccRCC scRNA-seq landscape, involving pRCC, chrRCC, CDRCC, and sarRCC. On the other hand, we used TCGA cohort to validate the clinical outcomes of identified heterogenic cell clusters, minimizing this limitation.

Finally, the cell-type-particular markers expression schema depicted in the present work may help better understand the TME heterogeneity and biological characteristics of nccRCCs. The markers of different subsets of cells unveiled in this work are nccRCC-specific (DEGs with corresponding normal-derived cells), and may be better markers in diagnosis or other biological experiments. In conclusion, our work provides a new understanding of TME in nccRCC and may contribute to the development of new efficient therapies for nccRCC.

## Materials and methods

### Tissue processing and 10x genomics sequencing

A total of ten tissue samples were acquired from four patients receiving radical nephrectomy at the Third Affiliated Hospital of the Second Military Medical University (Shanghai, China), involving primary and metastatic tumor sites, plus para-tumor normal kidney tissues. Pathologic and clinical information were maintained in the medical database by the Department of Urology (Table 1 and Fig. S4 and Supplementary files for pathological reports). All participating patients provided written informed consent. Ethical and operational approval of all research procedures was provided by the Scientific Research Review and Investigation Committee of the Third Affiliated Hospital of the Second Military Medical University ([2018]:No.012).

**Table 1:** Pathologic and clinical information of 10x Genomics sequencing samples.

Patient ID	Tissue site	Sample ID	Sex	Age	Pathology	TNM stage
D1	Primary site	chrRCC_P	Male	54	(Right Kidney) Chromophobe cell carcinoma with iliac bone metastases	Satge III
	Para-tumor site	chrRCC_N				
	Metastatic tumor site	chrRCC_B				
D2	Primary site	pRCC3	Male	61	(Right kidney) Papillary renal cell carcinoma, Type 1.Both ureter and vascular end (−)	Satge I
D3	Primary site	sarRCC_P	Male	39	(Right kidney) the tumor cells are distinctly heterogeneous, considering a renal cell carcinoma with sarcomatoid differentiation	Satge IV
	Para-tumor site	sarRCC_N				
	Metastatic tumor site	sarRCC_L				
D4	Primary site	cdRCC_P	Female	27	(Right kidney) Collecting duct carcinoma with hilar and retroperitoneal lymph node metastasis and spinal metastasis	Satge IV
	Metastatic tumor site	cdRCC_L				
	Metastatic tumor site	cdRCC_B				
D5	Primary site	pRCC1	Unknown	70	Papillary renal cell carcinoma	Stage I
	Para-tumor site	pRCC1N				
	Primary site	pRCC2				
	Para-tumor site	pRCC2N				

We dissolved the 0.75 mg/ml type I collagenase (Cat: SCR103, Sigma), 2 mg/ml type IV collagenase (Cat: C5138, Sigma), 0.2 mg/ml hyaluronidase I-S (Cat: H3506, Sigma), and 0.0025 mg/ml DNase IV (Cat: D5025, Sigma) in the 0.25% trypsin (Cat: R001100, Thermo Fisher Scientific) to form the final 10 ml of digestion medium. Fresh renal primary tumor, para-tumor and metastatic tissue specimens were obtained from surgical resection of nccRCC patients. Each tissue sample was processed into tiny pieces (<1 mm in radius) and then incubated with prepared digestion solution on a 37 °C shaker until they were fully digested. Cell debris and clumps were filtered through nylon meshes (40 μm, Corning). Subsequently, the cell pellet was centrifuged at 500 × *g* and 4 °C for 5 min and then was re-suspended in 2 ml ice red blood lysis buffer after discarding the supernatant. 5 min of incubation later at room temperature, ice phosphate buffer saline (PBS) was added to 10 ml, and the suspension was centrifuged at 4 °C and 300 × *g* for 5 min. After removing the supernatant, we used automatic cytometry (Luna) to count the cell number and calculated the concentration. The optimal cell concentration and the target capture number were supplied by https://www.10xgenomics.com/. Gel beads in emulsion (GEMs) were prepared and reverse transcription was performed immediately once the expected cell concentration and viability was acquired.

### Public datasets acquisition

Considering that relatively low incidence of nccRCC, we attempted to include all the nccRCC of scRNA-seq for the more comprehensive analysis. Thus, the public scRNA-seq count matrices for human fetal kidney and pRCC (pRCC1, pRCC1N, pRCC2, and pRCC2N) were respectively from Hochane et al. [63] and Young et al. [15]. The expression matrices and corresponding clinical data of the patients from TCGA KIRP and KICH datasets (Table 2) containing 210 pRCC and 64 chrRCC samples (excluding cases with incomplete information, Table S1), which was acquired from official website (http://xena.ucsc.edu/) of UCSC Xena.

**Table 2:** The baseline clinical characteristics of TCGA nccRCC patients (*n* = 274).

Characteristics	Abundance of CD8T_cluster3_cells		*p* value	Abundance of sarRCC_cells		*p* value	Abundance of chr_Macrophages		*p* value	Consensus cluster			*p* value
	Low (*n* = 137)	High (*n* = 137)		Low (*n* = 137)	High (*n* = 137)		Low (*n* = 137)	High (*n* = 137)		1 (*n* = 145)	2 (*n* = 69)	3 (*n* = 60)	
Age			0.223			0.010			0.190				0.620
<60	44	54		39	59		44	54		51	23	24	
≥60	91	82		97	76		92	81		93	46	34	
NA	2	1		1	2		1	2		1	0	2	
Gender			0.642			0.729			0.879				0.305
Female	40	36		39	37		38	38		36	24	16	
Male	96	98		95	99		99	95		107	44	43	
NA	1	3		3	1		0	4		2	1	1	
AJCC stage			<0.001			0.005			<0.001				<0.001
I–II	131	100		124	107		137	94		145	69	18	
III–IV	6	37		13	30		0	43		0	0	42	
TNM stage			<0.001			0.010			<0.001				<0.001
I–II	131	104		125	110		137	98		145	69	22	
III–IV	6	33		12	27		0	39		0	0	38	
Overall survival			0.009			0.020			0.009				0.033
−	133	121		132	122		133	121		137	66	51	
+	4	16		5	15		4	16		8	3	9	
Progression-free survival													
−	122	118	0.464	122	118	<0.001	125	115	0.067	129	62	49	0.284
+	15	19		15	119		12	22		16	7	11	

The detailed information of these TCGA nccRCC patients, see the “Materials and methods” and Table S1.

### Droplet-based scRNA-seq and raw data processing

Chromium Single-Cell 3′ Library, Gel Bead & Multiplex Kit and Chip Kit (10x Genomics) were used to obtain barcoded scRNA-seq libraries, which were then sequenced on the Illumina NovaSeq 6000, generating 150 bp paired-end reads and processed through CellRanger (version 3.0.2) to obtain the label of human genome (Grch38). All the final raw count matrices of unique molecular identifier (UMI) were imported into R program (version 3.5.2).

### Data quality control (QC) and cell type identification

A total of 11 tumor samples, four normal kidney sample from six patients. (Figs. S5–S7) were included in our study. The data quality control was assessed via Seurat (version 3.0.1) [64]. Cells with <200 or >6000 genes or with more than 10% mitochondrial-derived genes were filtered out because of the low-quality. Finally, a total of 32,374 cells were included to perform further analysis. We used the Harmony package (version 1.0) to minimize differences between batches. The top 30 principal components and the top 2000 variable genes were applied. The ScaleData function of Seurat was used to regress out the interference of sample source, UMI counts and mitochondrial gene proportions. We used the FindClusters function of Seurat (resolution =2) to identify major cell clusters and visualized them by 2D UMAP or tSNE. We listed all the markers of each main cell cluster through FindAllMarker function. Markers for identifying the primary cell types were obtained from the CellMarker [65] and PanglaoDB [66] datasets, plus previous studies [19, 62, 67]. The characteristic markers used for label were presented in Fig. S8. Meanwhile, we used the SingleR package [68] as the auxiliary tool to identify cell types for the sake of screening out optimal labeling by the combined methods.

### Immunofluorescence (IHC) of human nccRCC tissue

These nccRCCs tissue samples for scRNA-seq were also obtained form the Department of Pathology in our institution and performed IHC assay. To detect specific gene expression, the antibodies for IHC are as following: anti-CD163 (mouse, 1:100, servicebio, catalog no. GB14027), anti-TREM2 (rabbit, 1:100, Proteintech, catalog no. 27599–1-AP), anti-CD8 (rabbit, 1:1000, servicebio, catalog no. GB13068), anti-LAG3 (rabbit, 1:30, Abcam, ab209236). The number of double-positive cells in random 20 fields were counted and analyzed statistically.

### Tumor purity and immune infiltration analysis

ESTIMATE is a pipeline using expression matrix to generate scores for tumor purity and the infiltrating level of immune cells or stromal cells. All the main cell clusters were classified as Tumor_cells, Immune_cells and Others, and then the ESTIMATE scores were obtained from these three types of cells, knowing that this method can provide the consideration of cancer-related cells for single-cell transcriptomic analysis when combined with Seurat Package [69].

### Pseudotime trajectory analyses

In order to investigate the alternations of TME, we utilized Monocle 3 and Monocle 2, the R packages shared by Cao et al. and Qiu et al. for single-cell dramatic trajectories [70, 71]. In Monocle 2, highly changed genes were recognized by the built-in differential GeneTest function.

### Differential expression and pathway analyses

Seurat’s FindMarkers function was used to calculate DEGs. The log_2_ (fold change) value (0.8~2) was set appropriately and adj. *p* value < 0.01 was used as the cutoff threshold value. The DEGs were then processed by clusterProfiler package for KEGG enrichment analysis [72]. The adj. *p* value < 0.05 was considered markedly enriched for each pathway. GSEA was applied to explore the significantly enriched gene set for every specific cluster with *p* value < 0.05 of false discovery rate. GSVA method was used to evaluate pathway activity for each cell with default parameters.

### Intercellular communication analyses

iTALK (R package, version 0.1.0) was utilized to analyze the cell–cell communication as a calculation tool [73]. Knowing that this method integrates various differential analysis and visualization approaches, which annotates ligand-receptors into four categories, including cytokines, growth factors, immune checkpoints and others, we believed that this pipeline could be more suitable for the analysis of molecular intercellular communication among cells involving TME. The ligand-receptor pairs with *p* value < 0.05 were considered as significant interaction.

### Single-cell regulatory network analyses

To reveal the gene regulatory network of the nccRCC derived samples, we used the SCENIC package (version 1.1.2.1) reference to motifs database for GRNboost2 and RcisTarget (version 1.2.1). Potential co-expressed modules and regulons were obtained by GRNBoost2 from arboreto. Targeted modules and regulatory network activity were assessed by RcisTarget and AUCell (version 1.4.1), respectively [74, 75].

### InferCNV

We applied the InferCNV package with default parameters to explore the CNVs in renal tubular cells (proximal and DT cells and CD cells) and to identify real renal cancer cells and CSC. Normally derived renal tubular cells were classified as the control group.

### Validation in external RNA-seq and clinical data of TCGA

CIBERSORTx tool (R package version) was used to detect the relative abundance of cell types defined by our single-cell data in bulk RNA-seq database. The bulk RNA-seq data was normalized by log_2_(TPM + 1) before being loaded into CIBERSORTx analysis. After establishing the signature matrix, we performed the relative abundance analysis for all cell clusters. The patients were divided into two equal groups: 50% high and 50% low. The KM analysis was used by GraphPad Prism (version 7.0) for hazard ratio. To investigate whether the abundance of cell clusters was correlated with survival changed accompanied by the TNM stage progression, we used Mann–Whitney test and visualized the results by boxplot. Nomogram analysis was performed by “foreign” (version 0.8-78) and “rms” packages (version 6.0-1) for constructing the risk prediction model. In order to confirm the significance of the TME subtype, ConsensusClusterplus (version 1.46.0) was performed to divide KICH and KIRP (TCGA cohort) into three groups. One-way ANOVA was employed to assess whether the relative abundance of cell cluster in these three nccRCC subgroups presented significant diversities. We used the Tukey’s multiple comparison test to calculate differences between any two groups

## Supplementary information

Supplementary files

Supplementary Table 1

Emails for author changes.

Reproducibility checklist

## Data Availability

The 10X genomics data have been deposited (PRJCA003839) in the Genome Sequence Archive of Human in the BIG Data Center, Chinese Academy of Sciences under accession codes HRA000435 for that are publicly accessible at https://bigd.big.ac.cn/gsa-human.

## Code availability

All the codes can be obtained from https://github.com/JovannyChen/Non-clear-cell-renal-cell-carcinoma-a-single-cell-genomics-study as the reasonable request.
