# Supplementary material for: Heterogeneity of tumor microenvironment is associated with clinical prognosis of non-clear cell renal cell carcinoma: a single-cell genomics study
Source: Cell Death Dis. 2022 Jan 11;13(1):50. doi: 10.1038/s41419-022-04501-9 (PMC8752784; doi:10.1038/s41419-022-04501-9)
Supplement: Supplementary file 1 — Supplementary files [file 41419_2022_4501_MOESM1_ESM.pdf]

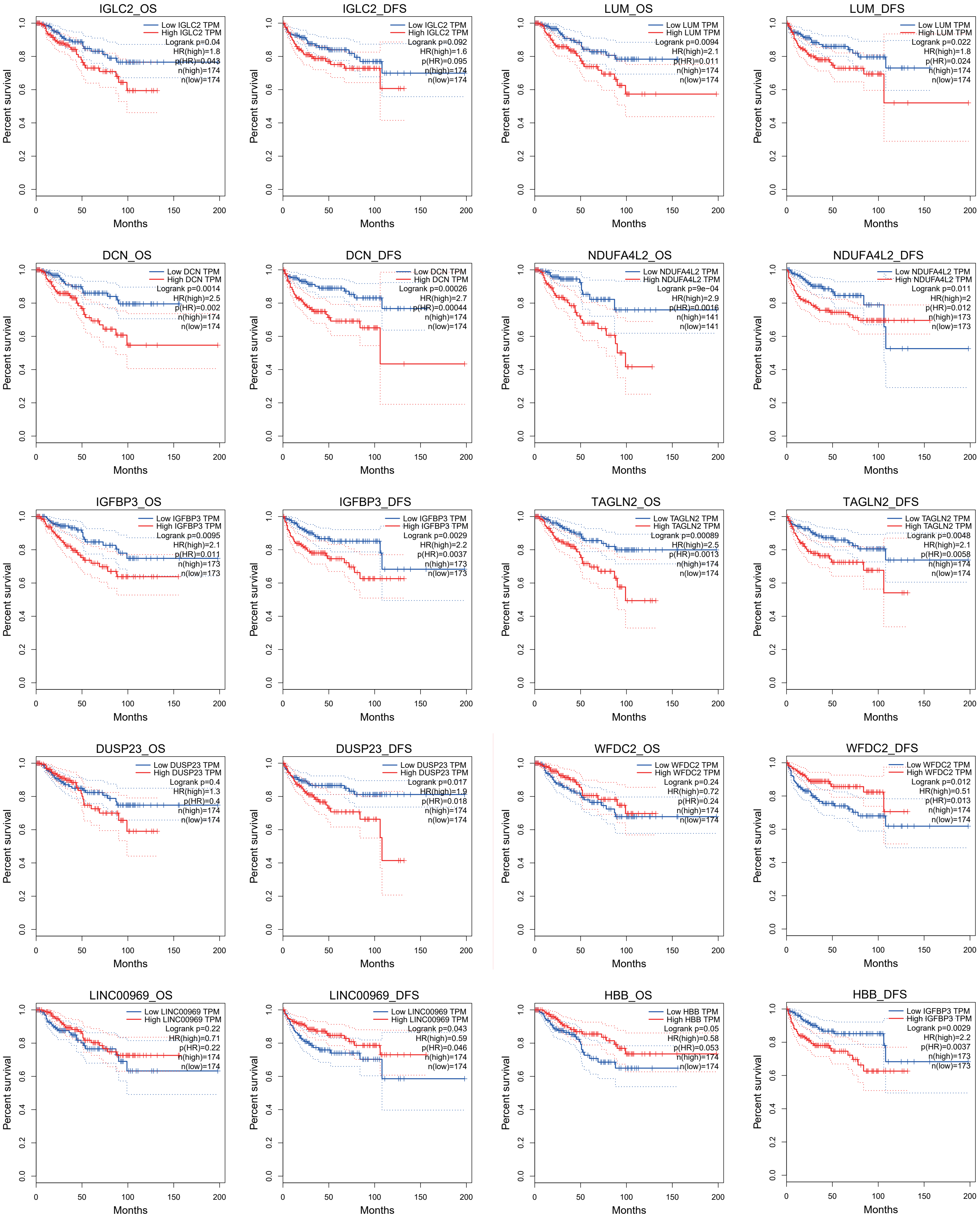


**Fig. S1 The survival curve for DEGs.**

**Figure S1**

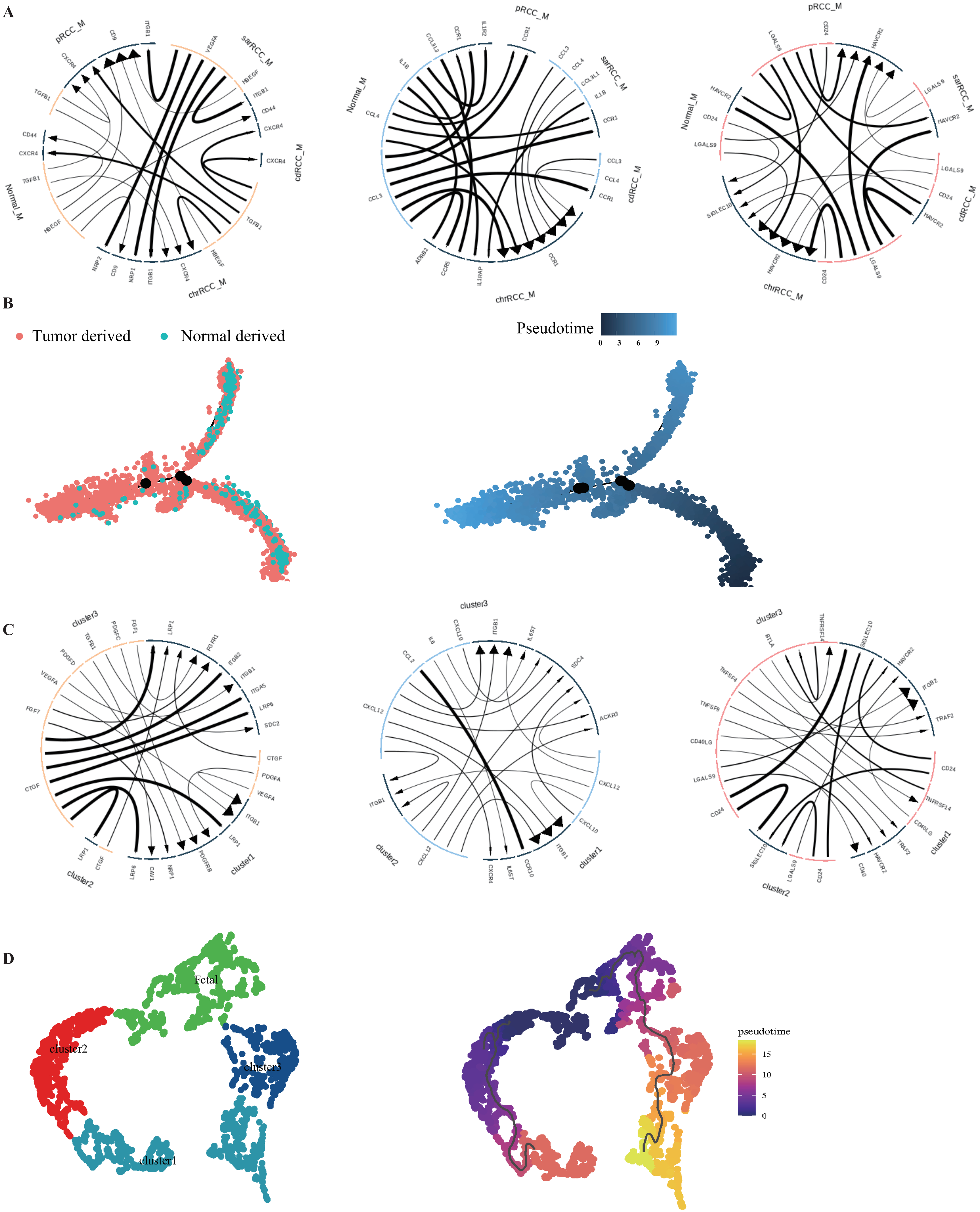


**Fig. S2** Cell crosstalk between different Macrophage clusters (A). Macrophages differentiation trajectory (B). Cell crosstalk between different Fibroblast clusters (C). Fibroblast differentiation trajectory (D).

**Figure S2**

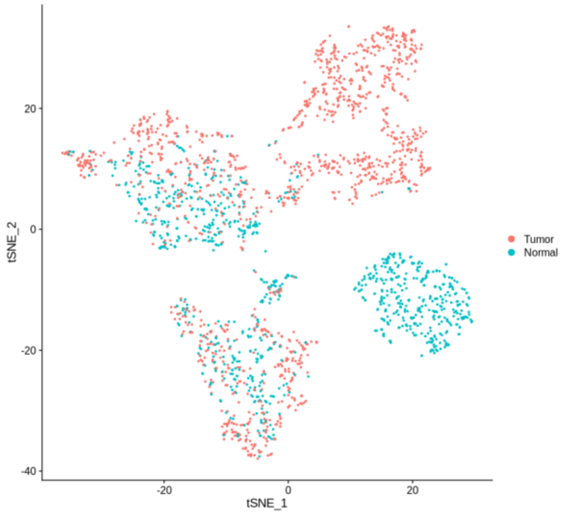


**Fig. S3** A total of 2217 cells were identified as fibroblasts, and mostly were tumor-derived.

**Figure S3**

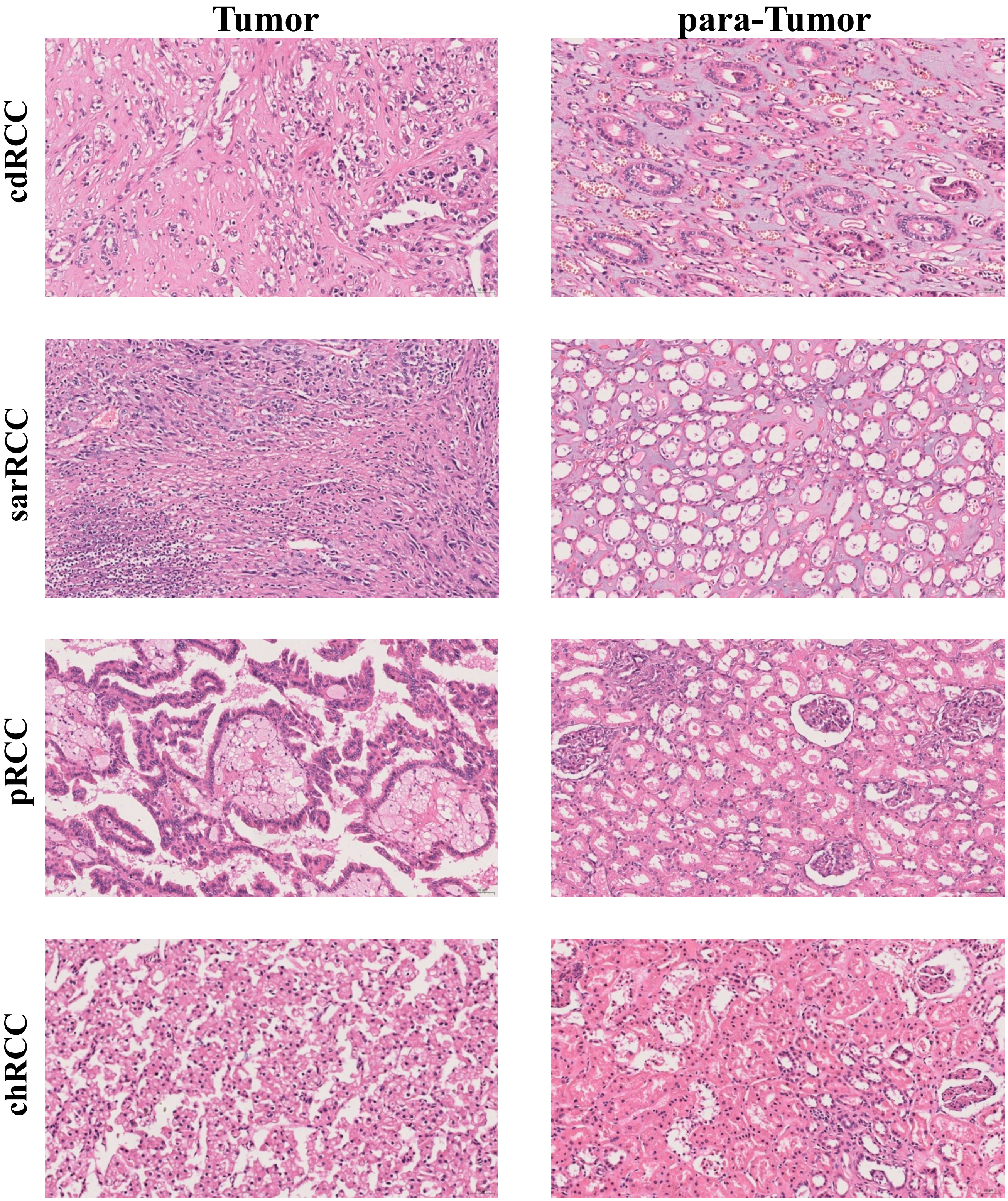


**Fig.S4 HE staining of nccRCC tissues and para-Tumor tissues from four patiens in our institution. (bar=50 μm)**

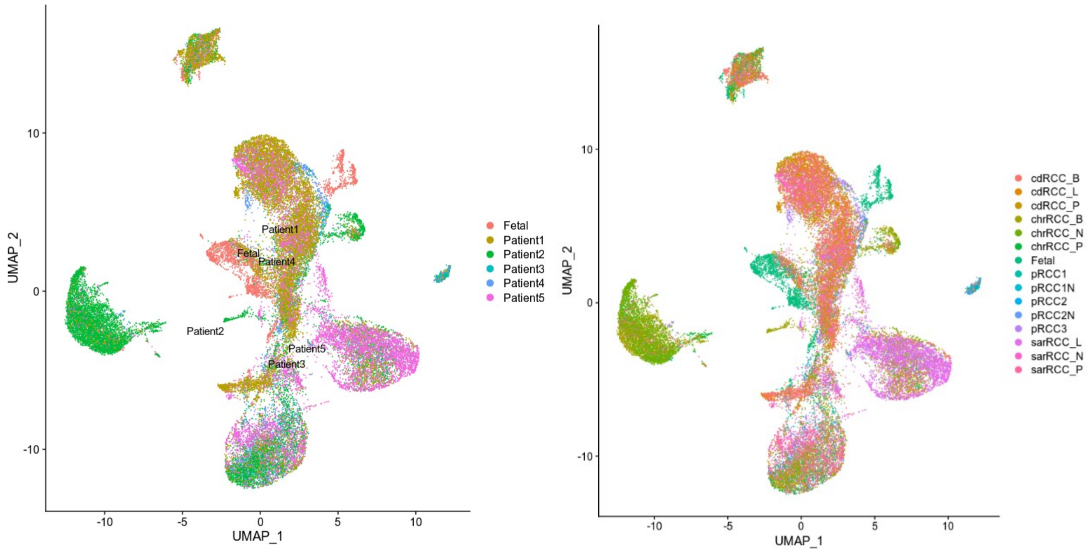


**Fig. S5** A total of 11 tumor samples, 3 para-tumor samples and 1 fetal kidney sample from 5 patients and 1 embryonic kidney.

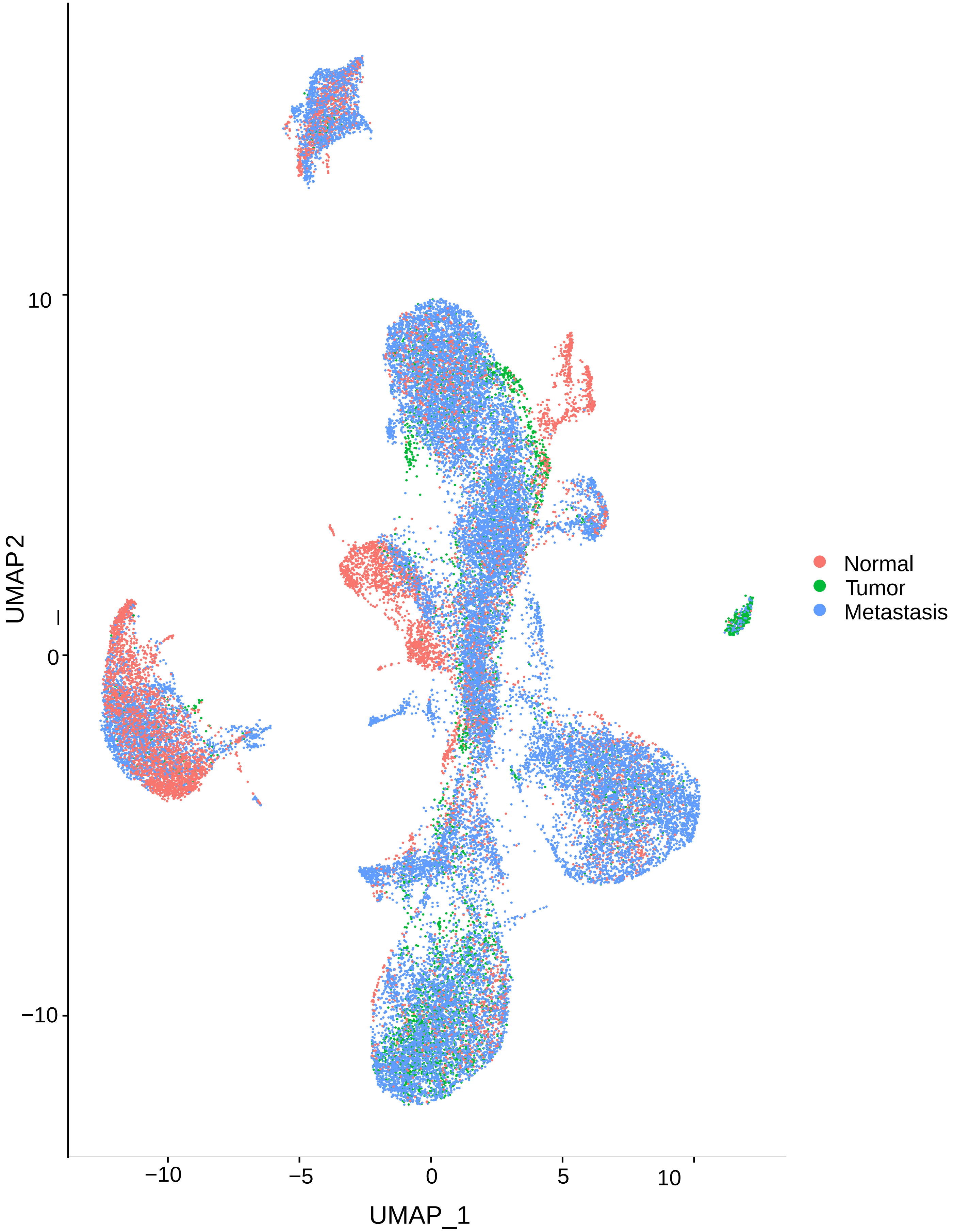


**Fig. S6** Cells resource from different samples.

**Figure S6**

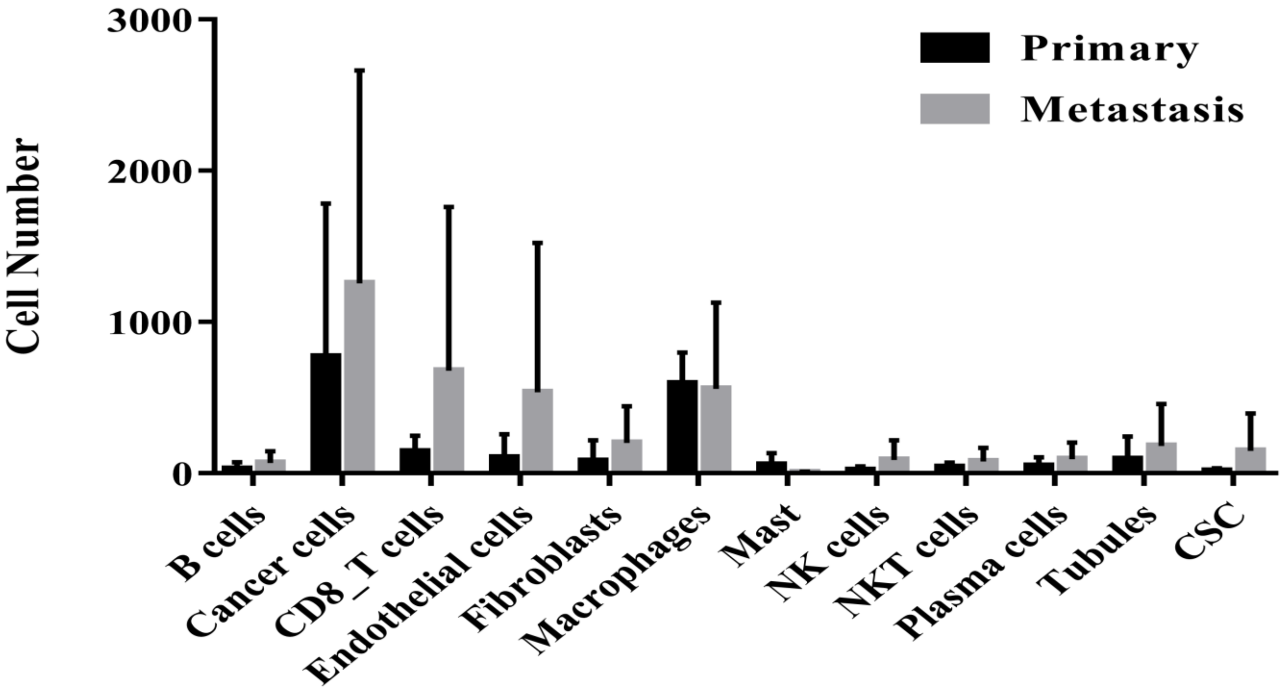


**Fig.S7 Cell number of each cell type in primary and metastatic site**

## B cells

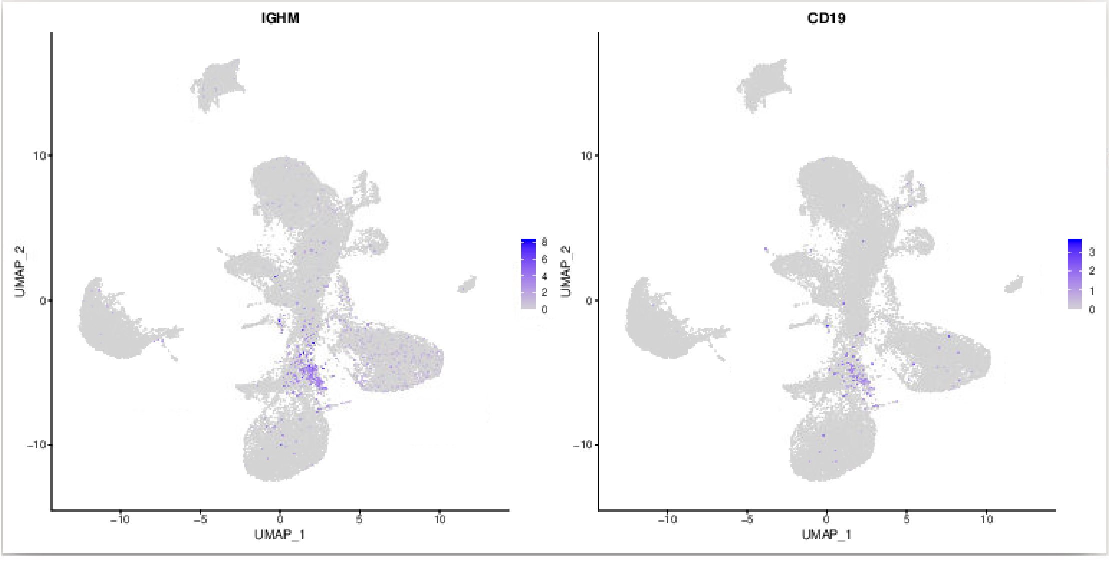


## CD8+ T cells

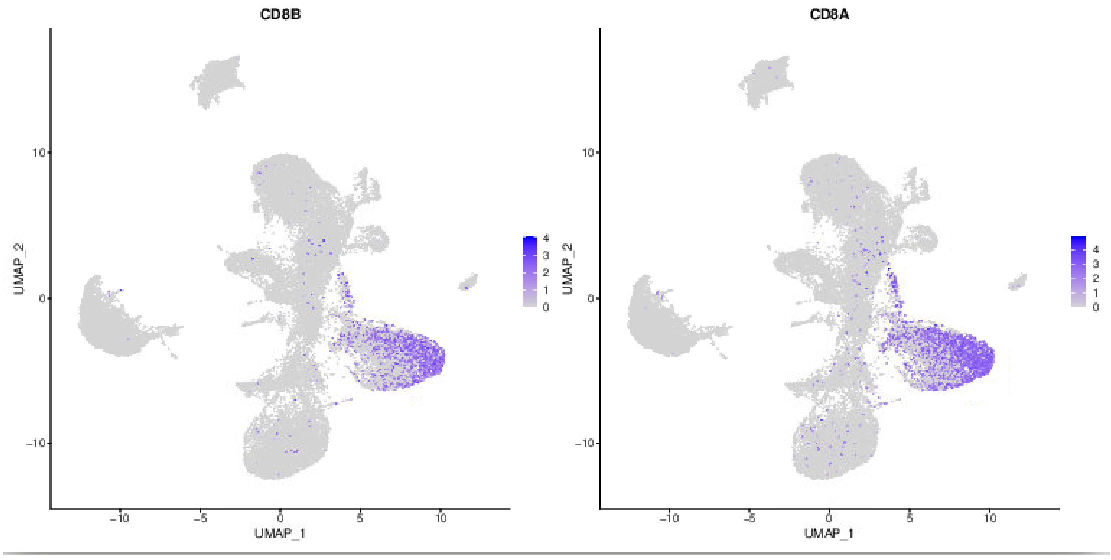


## CSC

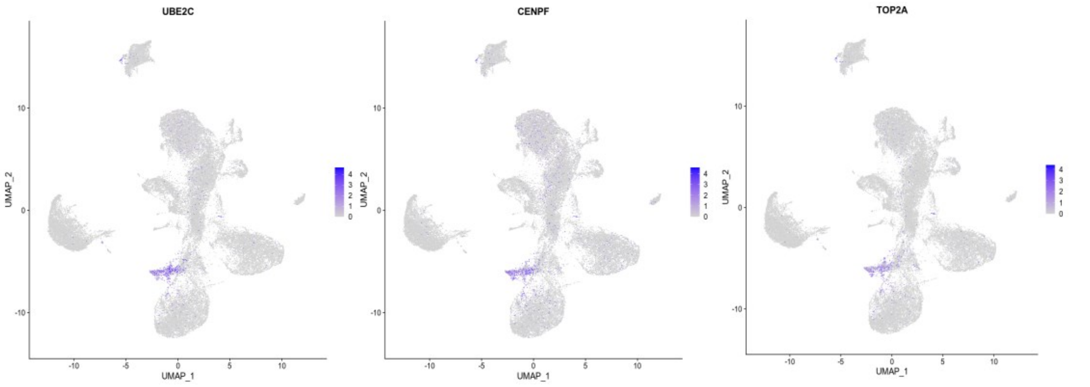


**Figure S8**

## Cancer cells

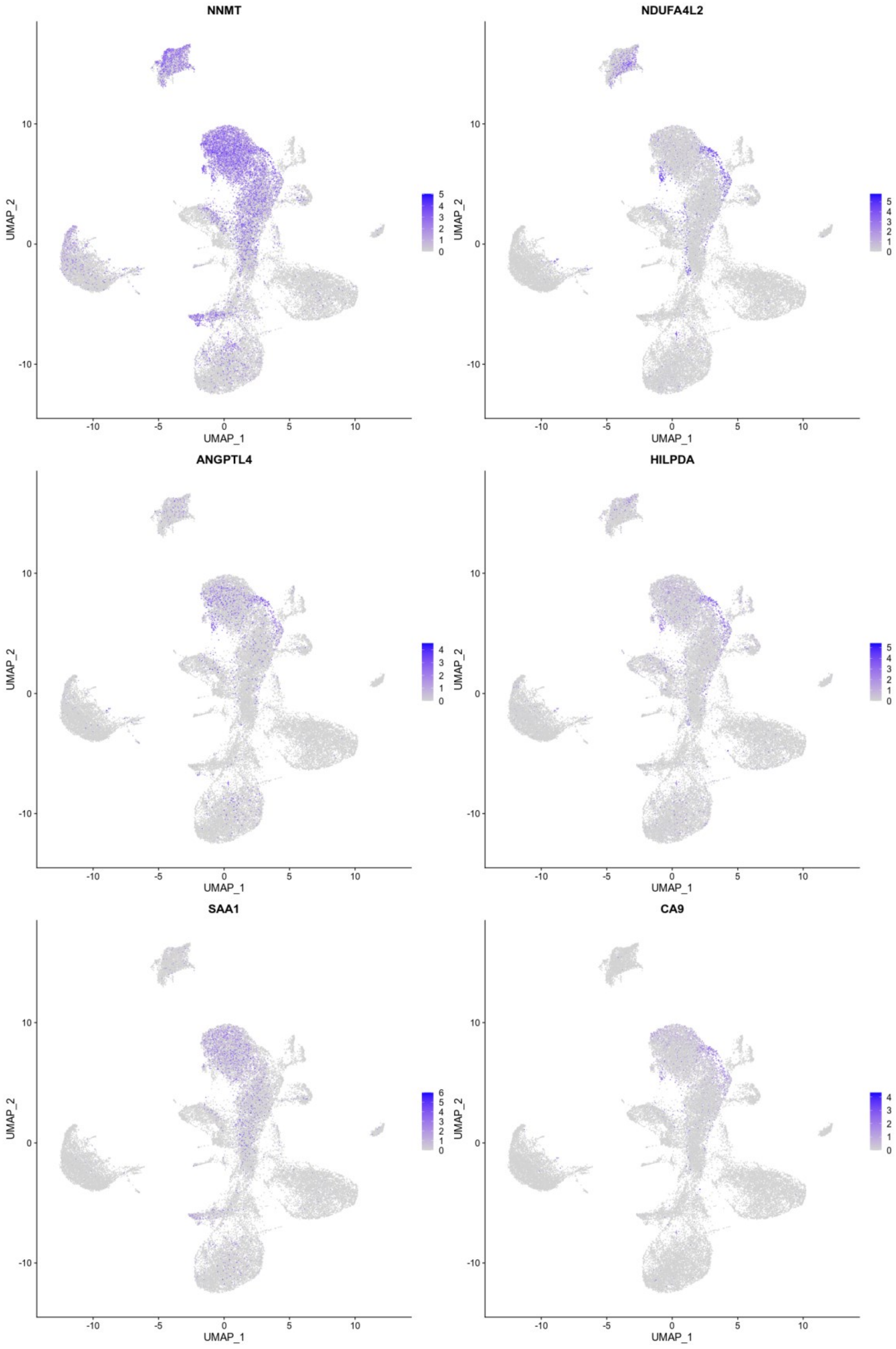


Figure S8

## Collecting duct cells

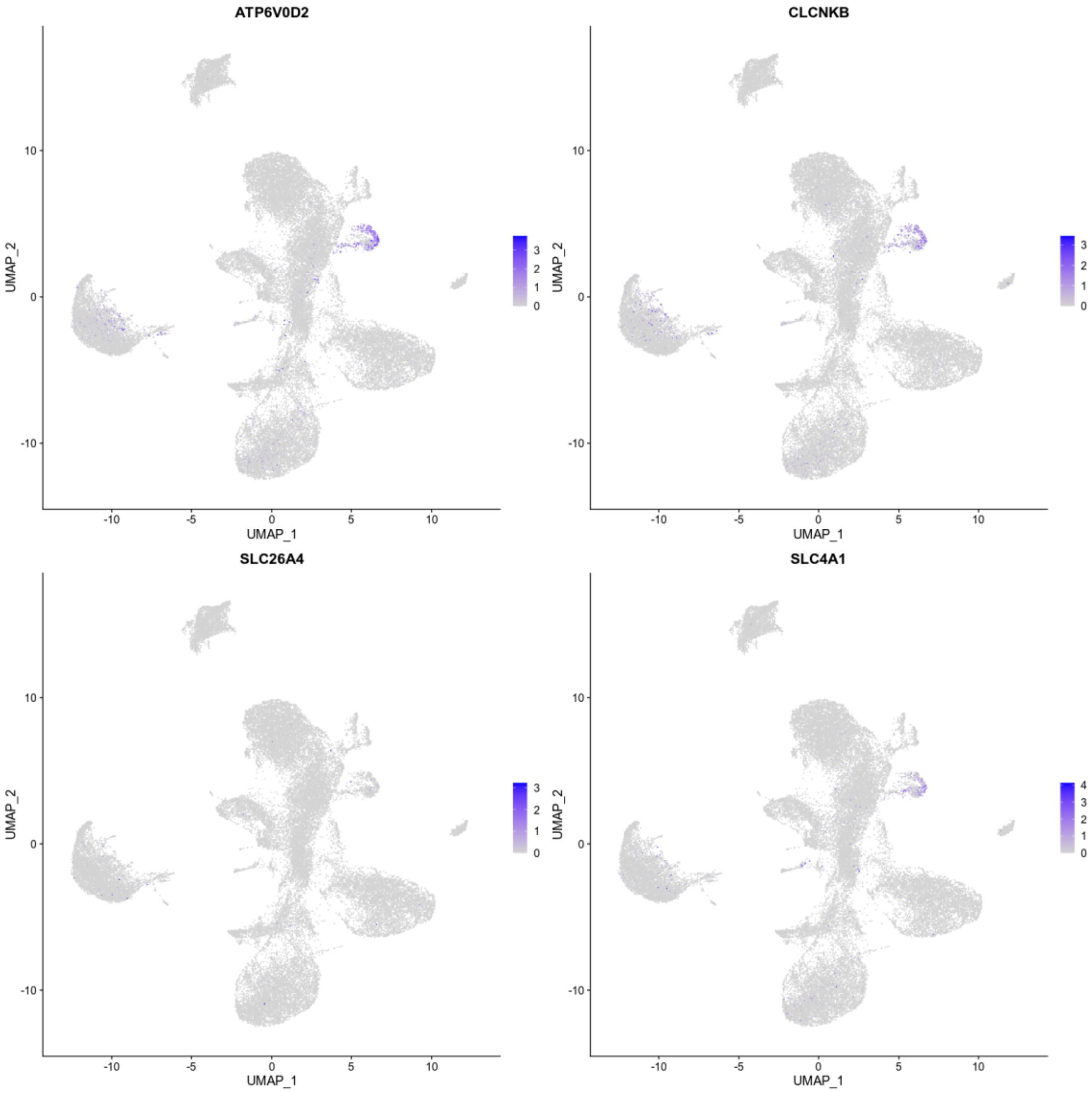


## Distal tubule cells

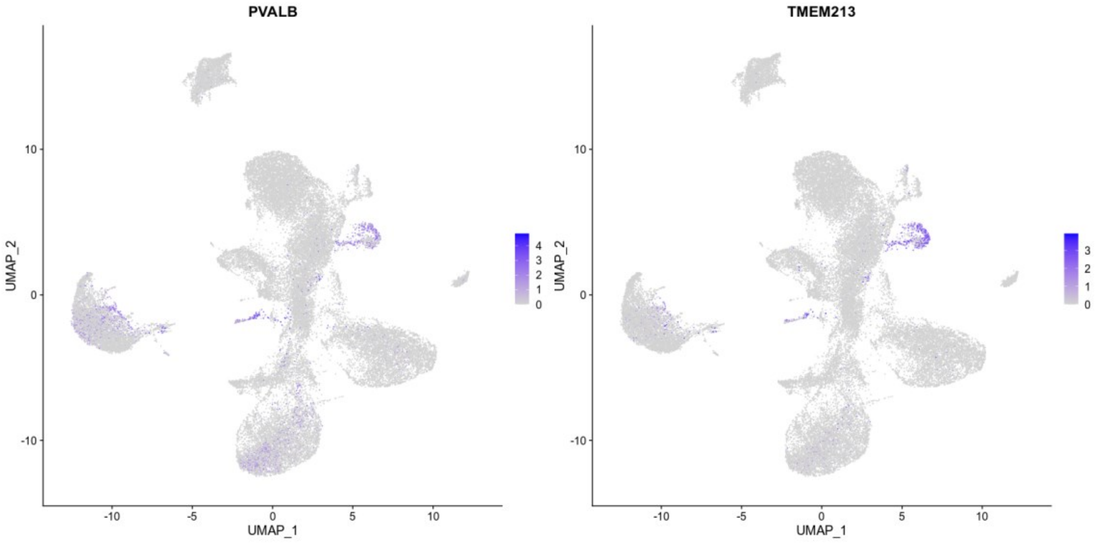


**Figure S8**

## Endothelial cells

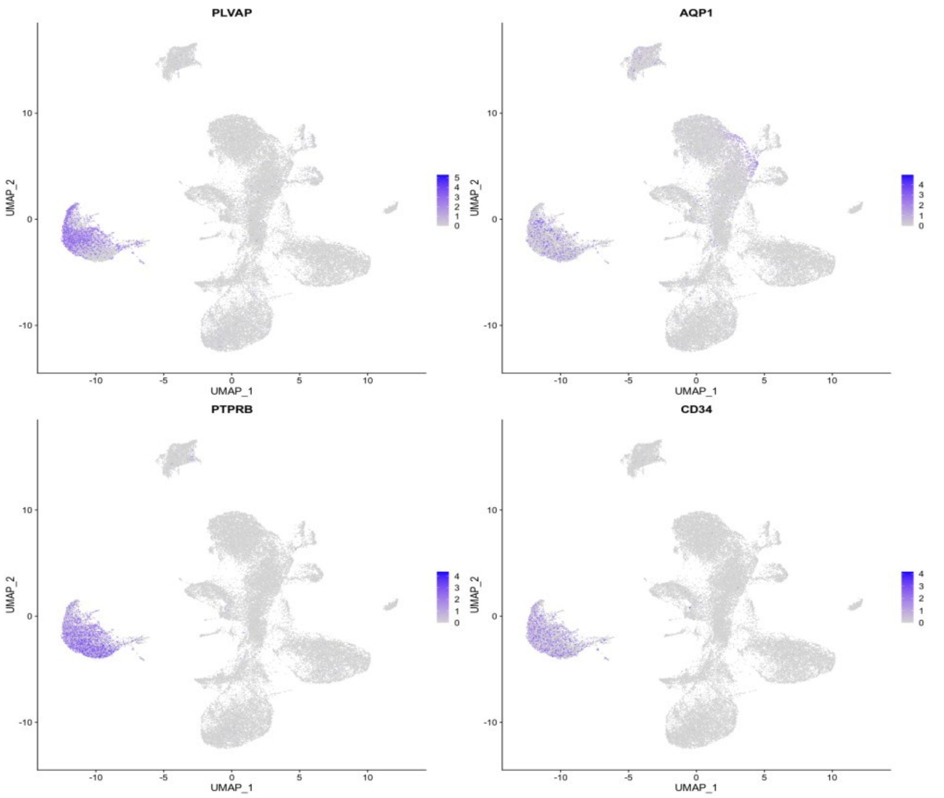


## Fibroblasts

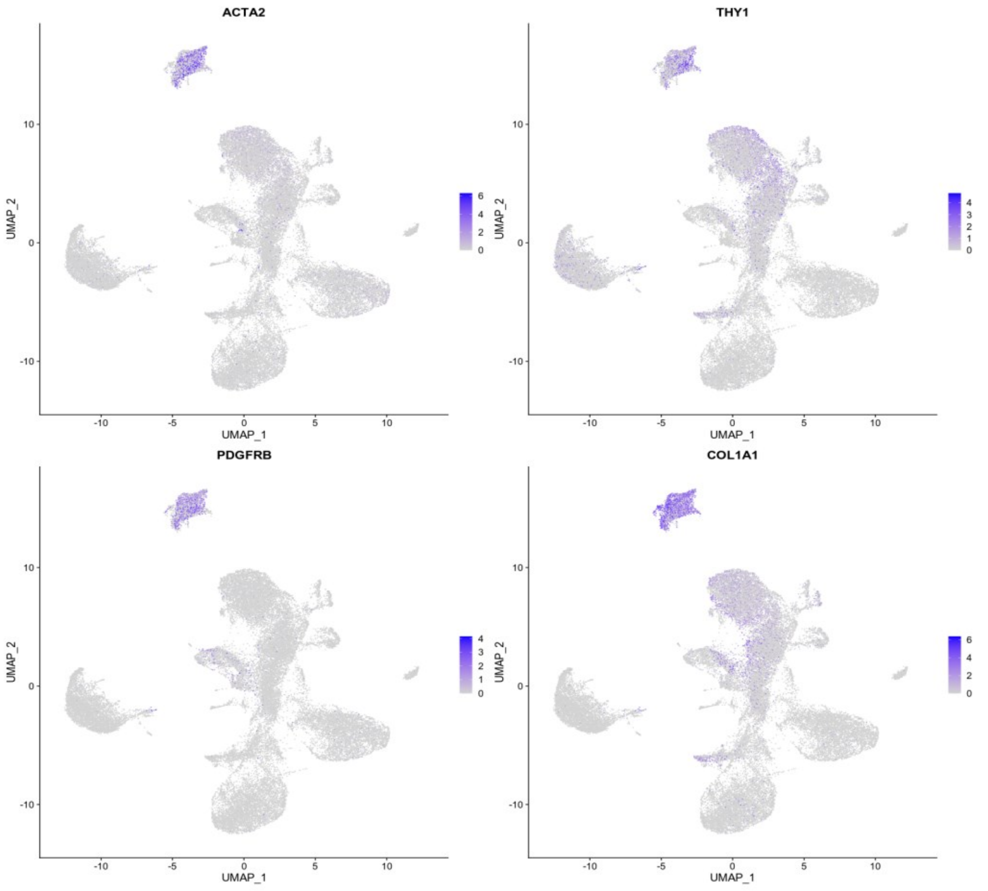


**Figure S8**

## Kidney progenitor cells

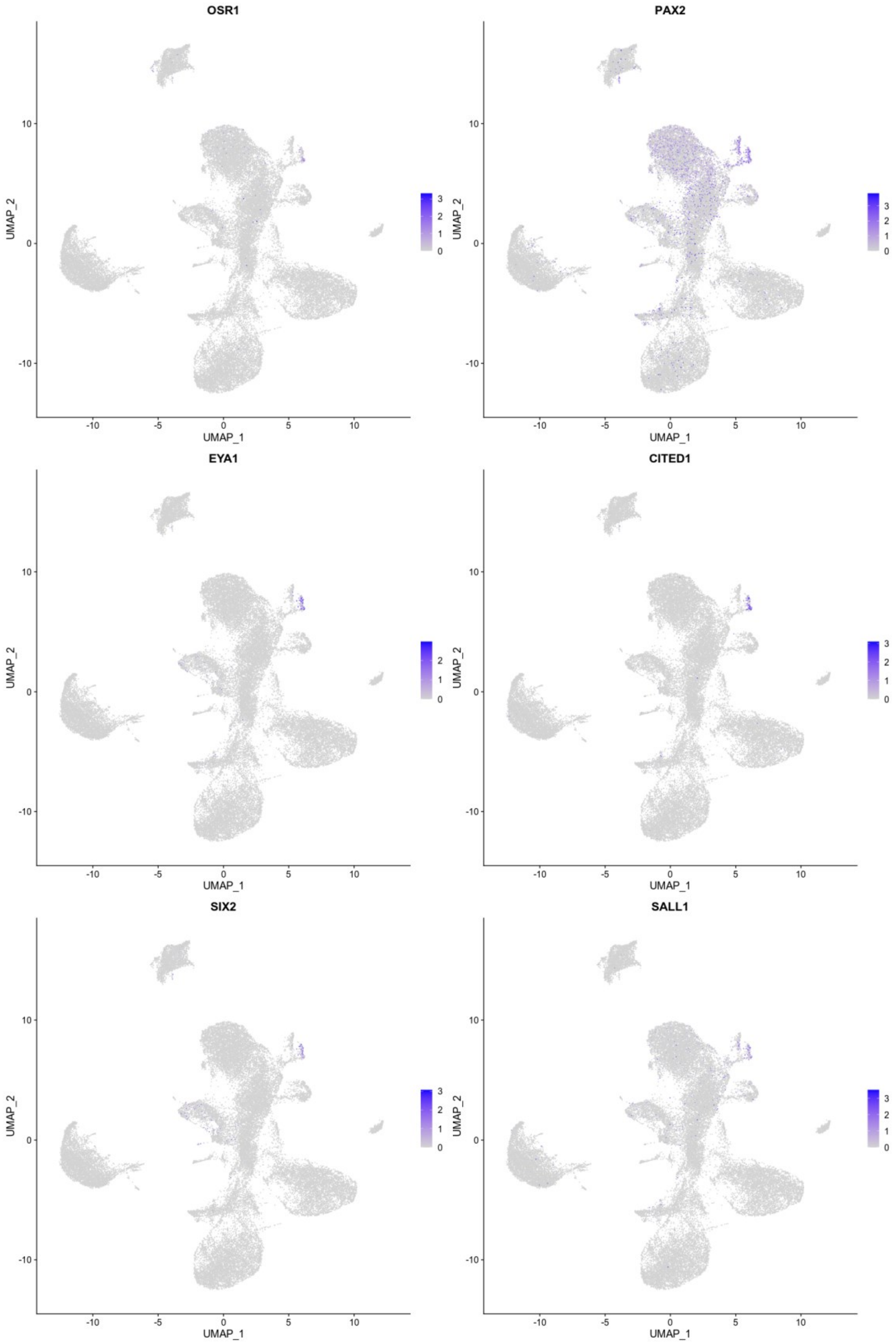


**Figure S8**

## Mast

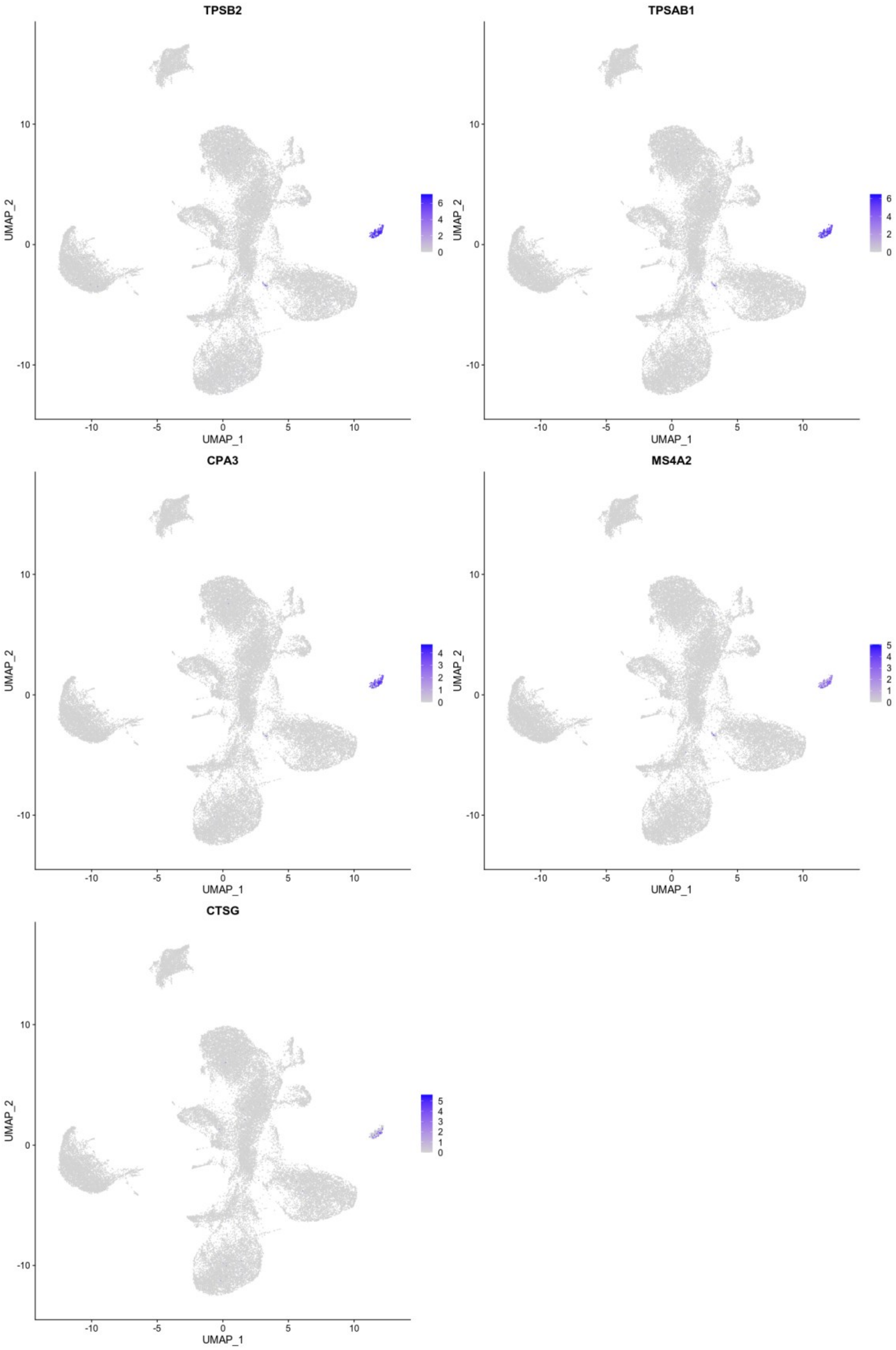


**Figure S8**

## Macrophages

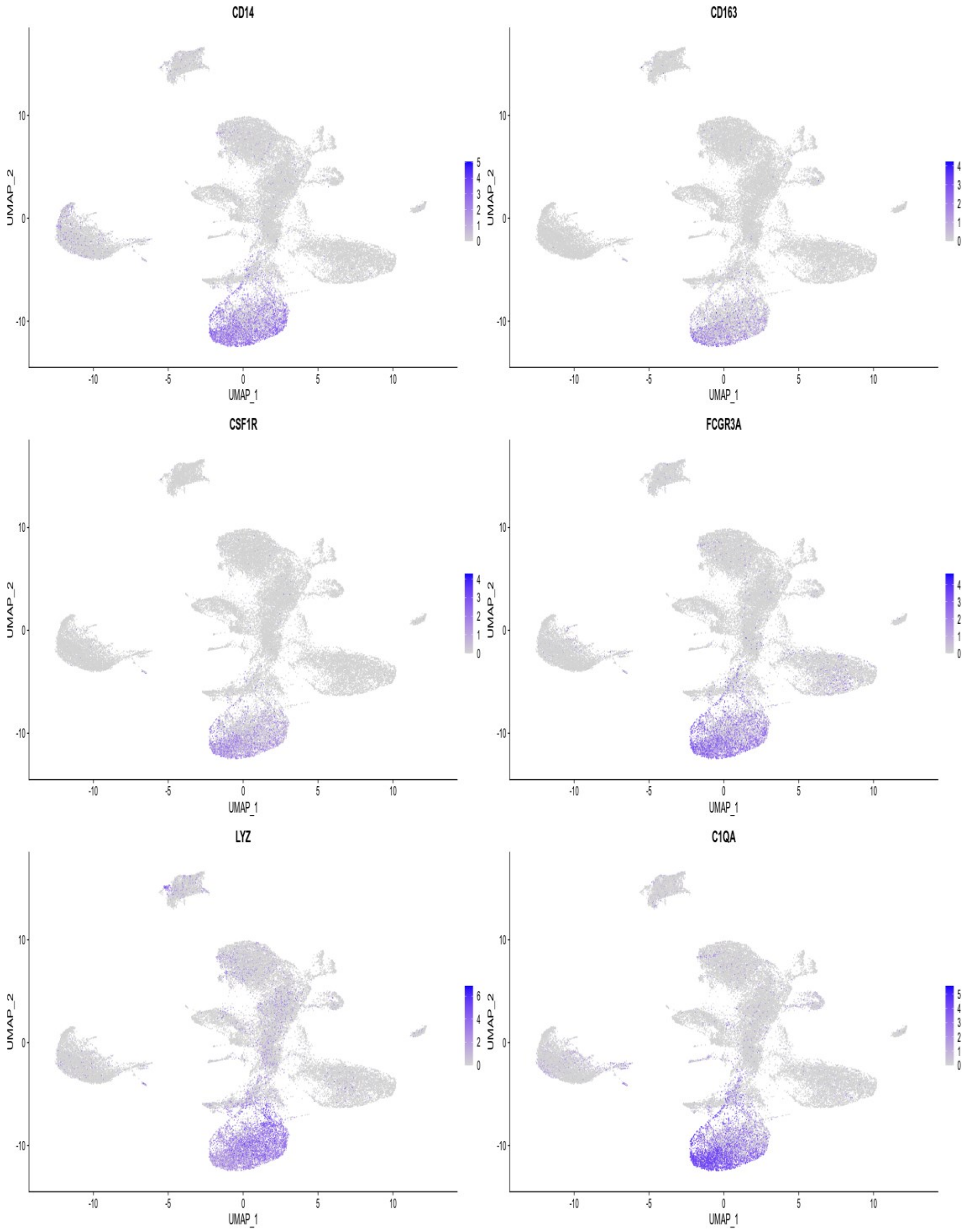


Figure S8

## NK cells

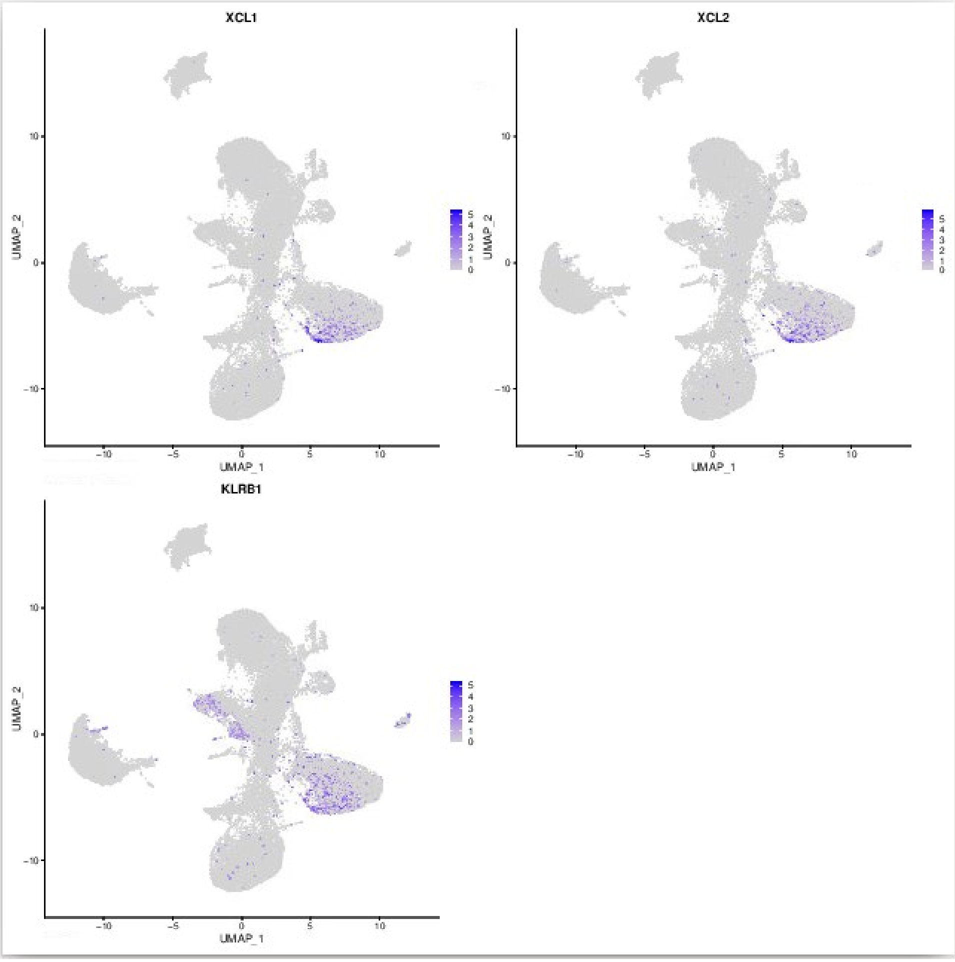


## NKT cells

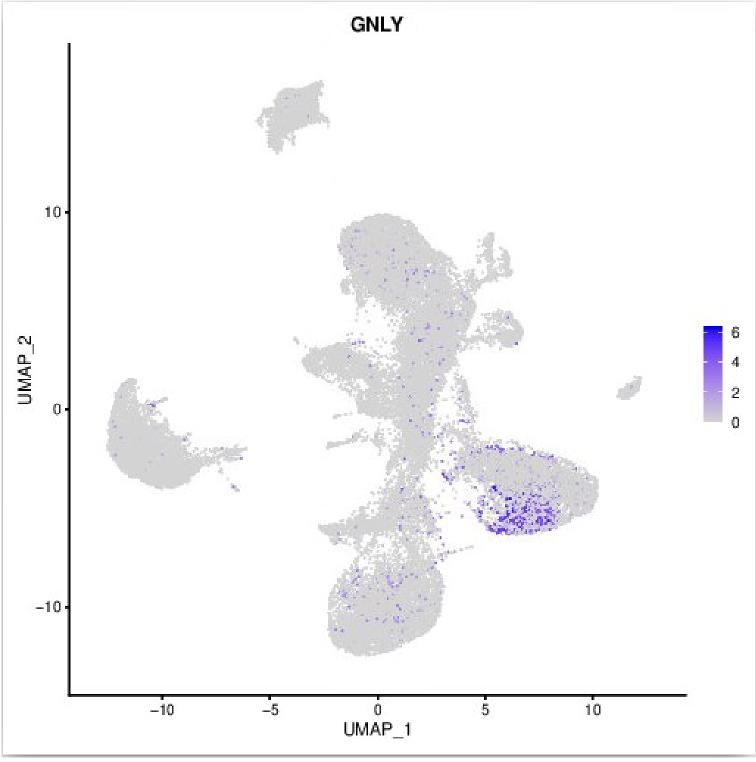


**Figure S8**

## Proximal tubule cells

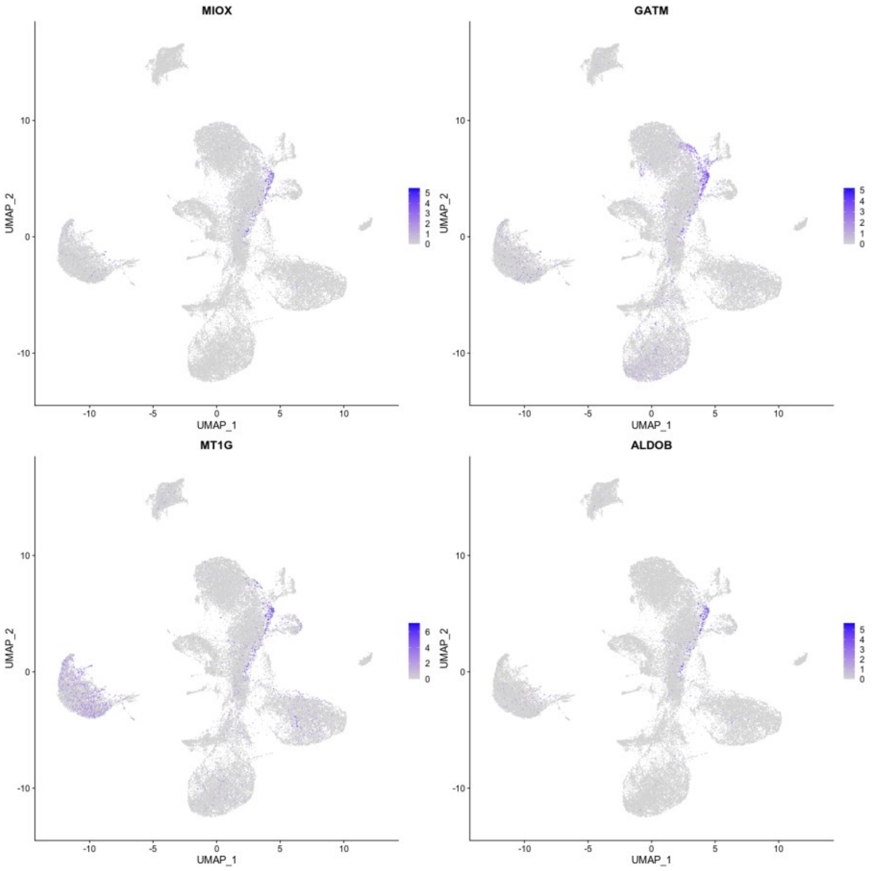


## Plasmas

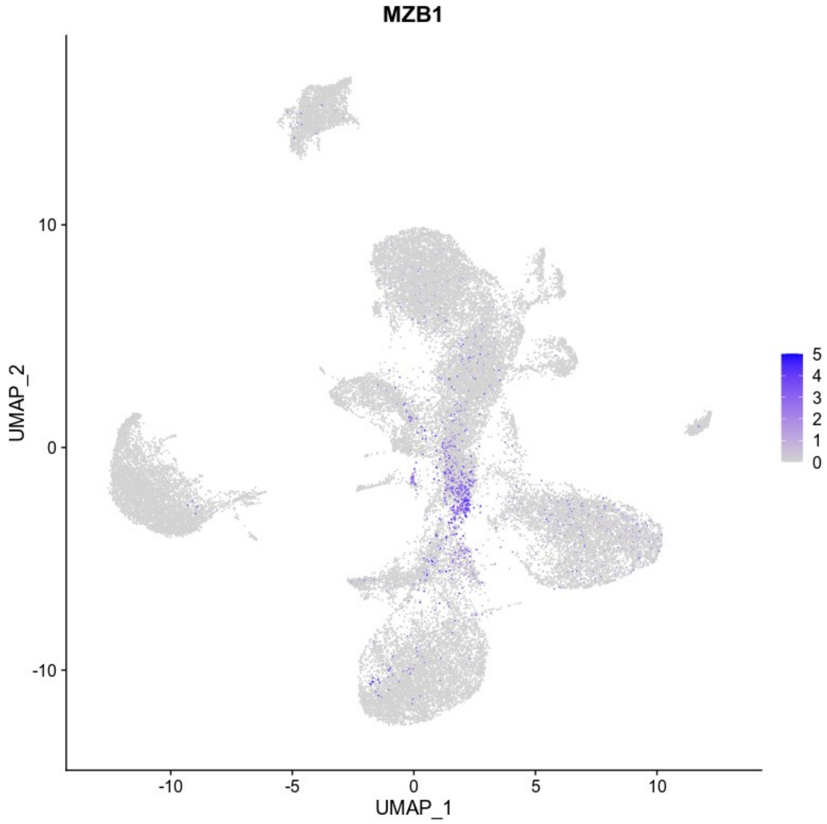


**Figure S8**

**Podocytes**

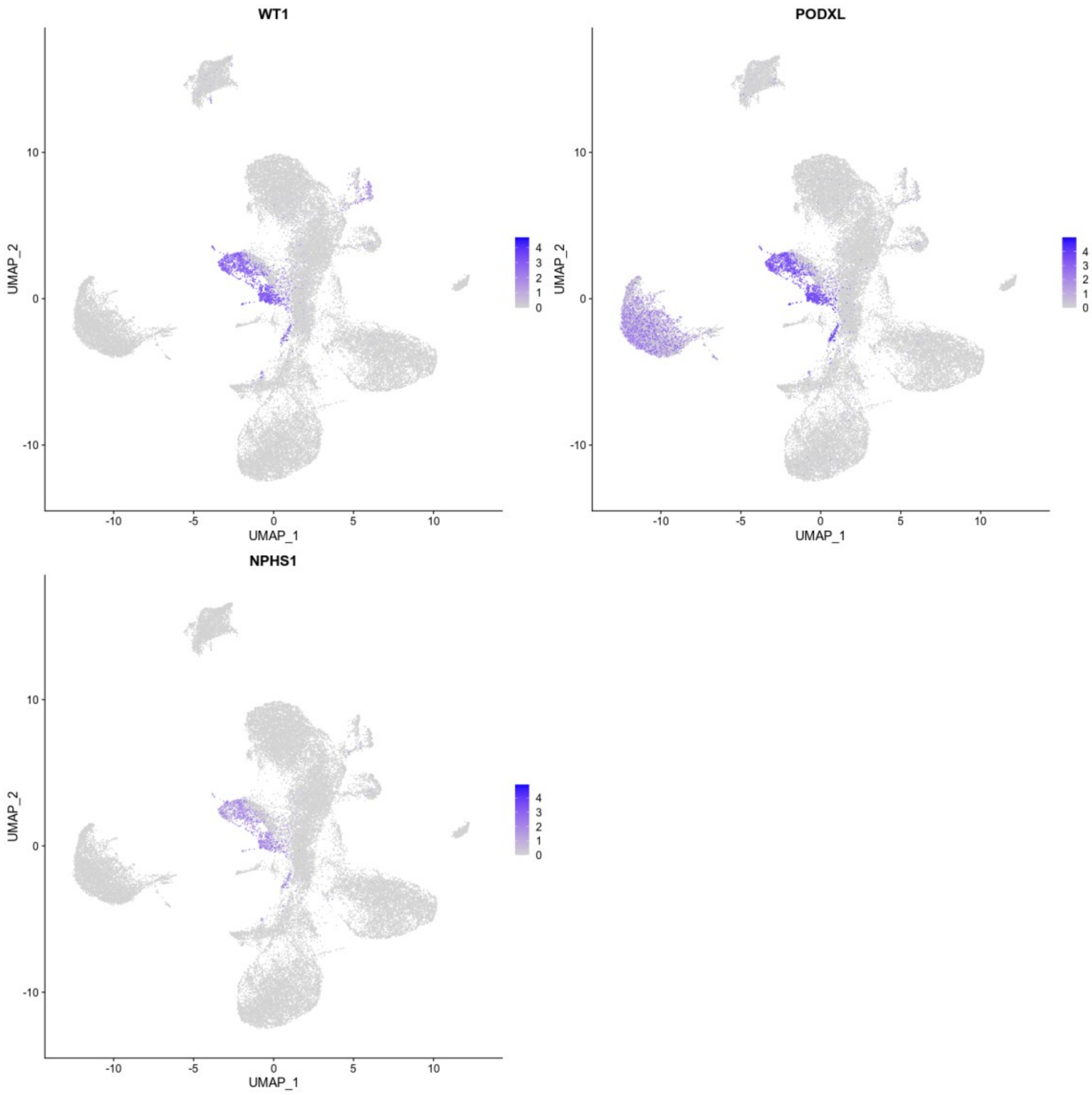


**Fig. S8** The characteristic markers used for label.
