# Supplementary material for: Heterogeneity of tumor microenvironment is associated with clinical prognosis of non-clear cell renal cell carcinoma: a single-cell genomics study
Source: Cell Death Dis. 2022 Jan 11;13(1):50. doi: 10.1038/s41419-022-04501-9 (PMC8752784; doi:10.1038/s41419-022-04501-9)
Supplement: Supplementary file 3 — Emails for author changes. [file 41419_2022_4501_MOESM3_ESM.pdf]

主　题:	Re: Author list change	
发件人:	"Chen Wen-jin" <chenwenjin@smmu.edu.cn>	2021-12-24 21:32:39
收件人:	"崔心刚" <cuixingang@smmu.edu.cn>	

OK, I agree with the final author list and order, including additions and deletions.

-----原始邮件-----
发件人:"崔心刚" <cuixingang@smmu.edu.cn>
发送时间:2021-12-24 19:28:06 (星期五)
收件人: chenwenjin@smmu.edu.cn, panxiuwu@126.com, vit_c@126.com, xuda@smmu.edu.cn, jiaxinchan@smmu.edu.cn, ye910@126.com, gansishun20101111@163.com, Chinacaohao@syphu.edu.cn, 15900611435@126.com, zuoli@njmu.edu.cn, davysky@163.com, doctor_zhanghao@163.com, geoff.ghy@126.com, brilliant212@163.com
抄送:
主题: Author list change

Colleagues and co-authors,

Our recent manuscript for ***Cell Death and Disease*** has been accepted (Heterogeneity of tumor microenvironment is associated with clinical prognosis of non-clear-cell renal cell carcinoma: a single-cell genomics study, CDDIS-21-2730RRR).

This following is the final author list and order, including additions and deletions. **Please reply whether agree or not as soon as possible.**

Heterogeneity of tumor microenvironment is associated with clinical prognosis of non-clear-cell renal cell carcinoma: a single-cell genomics study

Running title: scRNA presents nccRCC TME profile correlating with prognosis

Chen Wen-jin[1]†, Cao Hao[2,6†], Cao Jian-wei[3]†, Zuo Li[4]†, Qu Fa-jun[3], Xu Da[1], Zhang Hao[5], Gong Hai-yi[5], Chen Jia-xin[1], Ye Jian-qing[1], Gan Si-shun[1], Zhou Wang[1,3], Zhu Da-wei[4*], Pan Xiu-Wu[1,3]†, Cui Xin-gan

1. Department of Urology, The Third Affiliated Hospital of Second Military Medical University, 700 North Moyu Road, Shanghai 201805, China. chenwenjin@smmu.edu.cn (Chen Wen-jin); panxiuwu@126.c

(Xu Da); jiaxinchan@smmu.edu.cn (Chen Jia-xin); ye910@126.com (Ye Jian-qing); gansishun20101111@163.com (Gan Si-shun); brilliant212@163.com (Zhou Wang); cuixingang@smmu.edu.cn (Cui X

2. School of Life Science and Biopharmaceutics, Shenyang Pharmaceutical University, Shenyang 110016, Chinacaohao@syphu.edu.cn (Cao Hao)

3. Department of Urology, Xinhua Hospital, Shanghai Jiaotong University, School of Medicine, 1665 Kongjiang Road, Shanghai 200092, China. vit_c@126.com (Cao Jian-wei); 15900611435@126.com (Q

4. Department of Urology, the Affiliated Changzhou No. 2 People's Hospital of Nanjing Medical University, 29 Xinglong Road, Changzhou, Jiangsu 213000, China. zuoli@njmu.edu.cn (Zuo Li); davysky@

5. Department of Orthopedic Oncology, Changzheng Hospital of Second Military Medical University, 415 Fengyang Road, Shanghai 200003, China. doctor_zhanghao@163.com (Zhang Hao); geoff.ghy@1

6. Peking-Tsinghua Center for Life Sciences, TsinghuaUniversity, 100084 Beijing, China

† These authors contributed equally to this work and should be considered as co-first authors

*** Correspondence to:** Prof. Cui Xin-gang, M.D, Department of Urology, The Third Affiliated Hospital of Second Military Medical University, 700 North Moyu Road, Shanghai 201805, China; Department

Jiaotong University, School of Medicine, 1665 Kongjiang Road, Shanghai 200092, China. Email address: cuixingang@smmu.edu.cn. Tel.: +86 02181887661; fax: +86 02181887661. Pan Xiu-Wu, M.D, Depa

Hospital of Second Military Medical University, 700 North Moyu Road, Shanghai 201805, China; Department of Urology, Xinhua Hospital, Shanghai Jiaotong University, School of Medicine, 1665 Kongjian

address: panxiuwu@126.com. Zhu Da-wei, Department of Urology, the Affiliated Changzhou No. 2 People's Hospital of Nanjing Medical University, 29 Xinglong Road, Changzhou, Jiangsu 213000, China. E

Cui Xin-gang

主　题:	Re: Author list change
发件人:	caohao <caohao@syphu.edu.cn> 2021-12-24 20:14:14
收件人:	"cuixingang@smmu.edu.cn" <cuixingang@smmu.edu.cn>

OK, I agree with the final author list and order, including additions and deletions

caohao

caohao@syphu.edu.cn

On 12/24/2021 19:33，崔心刚<cuixingang@smmu.edu.cn> wrote：

Colleagues and co-authors,

Our recent manuscript for ***Cell Death and Disease*** has been accepted (Heterogeneity of tumor microenvironment is associated with clinical prognosis of non-clear-cell renal cell carcinoma: a single-cell genomics study, CDDIS-21-2730RRR).

This following is the final author list and order, including additions and deletions. **Please reply whether agree or not as soon as possible.**

Heterogeneity of tumor microenvironment is associated with clinical prognosis of non-clear-cell renal cell carcinoma: a single-cell genomics study

Running title: scRNA presents nccRCC TME profile correlating with prognosis

Chen Wen-jin[1]†, Cao Hao[2,6†], Cao Jian-wei[3]†, Zuo Li[4]†, Qu Fa-jun[3], Xu Da[1], Zhang Hao[5], Gong Hai-yi[5], Chen Jia-xin[1], Ye Jian-qing[1], Gan Si-shun[1], Zhou Wang[1,3], Zhu Da-wei[4*], Pan Xiu-Wu[1,3]†, Cui Xin-gang

1. Department of Urology, The Third Affiliated Hospital of Second Military Medical University, 700 North Moyu Road, Shanghai 201805, China. chenwenjin@smmu.edu.cn (Chen Wen-jin); panxiuwu@126.co

(Xu Da); jiaxinchan@smmu.edu.cn (Chen Jia-xin); ye910@126.com (Ye Jian-qing); gansishun20101111@163.com (Gan Si-shun); brilliant212@163.com (Zhou Wang); cuixingang@smmu.edu.cn (Cui Xi

2. School of Life Science and Biopharmaceutics, Shenyang Pharmaceutical University, Shenyang 110016, Chinacaohao@syphu.edu.cn (Cao Hao)

3. Department of Urology, Xinhua Hospital, Shanghai Jiaotong University, School of Medicine, 1665 Kongjiang Road, Shanghai 200092, China. vit_c@126.com (Cao Jian-wei); 15900611435@126.com (Qu

4. Department of Urology, the Affiliated Changzhou No. 2 People's Hospital of Nanjing Medical University, 29 Xinglong Road, Changzhou, Jiangsu 213000, China. zuoli@njmu.edu.cn (Zuo Li); davysky@1

5. Department of Orthopedic Oncology, Changzheng Hospital of Second Military Medical University, 415 Fengyang Road, Shanghai 200003, China. doctor_zhanghao@163.com (Zhang Hao); geoff.ghy@12

6. Peking-Tsinghua Center for Life Sciences, TsinghuaUniversity, 100084 Beijing, China

† These authors contributed equally to this work and should be considered as co-first authors

*** Correspondence to:** Prof. Cui Xin-gang, M.D, Department of Urology, The Third Affiliated Hospital of Second Military Medical University, 700 North Moyu Road, Shanghai 201805, China; Department o

Jiaotong University, School of Medicine, 1665 Kongjiang Road, Shanghai 200092, China. Email address: cuixingang@smmu.edu.cn. Tel.: +86 02181887661; fax: +86 02181887661. Pan Xiu-Wu, M.D, Depar

Hospital of Second Military Medical University, 700 North Moyu Road, Shanghai 201805, China; Department of Urology, Xinhua Hospital, Shanghai Jiaotong University, School of Medicine, 1665 Kongjiang

address: panxiuwu@126.com. Zhu Da-wei, Department of Urology, the Affiliated Changzhou No. 2 People's Hospital of Nanjing Medical University, 29 Xinglong Road, Changzhou, Jiangsu 213000, China. Em

Cui Xin-gang

主　题:	Re: Author list change	
发件人:	"第二军医大学第三附属医院（东方肝胆外科医院）" <vit_c@126.com>	2021-12-24 20:18:04
收件人:	cuixingang <cuixingang@smmu.edu.cn>	

OK, I agree with the final author list and order, including additions and deletions

---- 回复的原邮件 ----

发件人	崔心刚<cuixingang@smmu.edu.cn>
日期	2021年12月24日 19:28
收件人	chenwenjin@smmu.edu.cn<chenwenjin@smmu.edu.cn>、panxiuwu@126.com<panxiuwu@126.com>、vit_c@126.com<vit_c@126.com>、xuda@smmu.edu.cn<xuda@smmu.edu.cn>、jiaxinchan@smmu.edu.cn<jiaxinchan@smmu.edu.cn>、ye910@126.com<ye910@126.com>、gansishun20101111@163.com<gansishun20101111@163.com>、Chinacaohao@syphu.edu.cn<Chinacaohao@syphu.edu.cn>、15900611435@126.com<15900611435@126.com>、zuoli@njmu.edu.cn<zuoli@njmu.edu.cn>、davysky@163.com<davysky@163.com>、doctor_zhanghao@163.com<doctor_zhanghao@163.com>、geoff.ghy@126.com<geoff.ghy@126.com>、brilliant212@163.com<brilliant212@163.com>
主题	Author list change

Colleagues and co-authors,

Our recent manuscript for ***Cell Death and Disease*** has been accepted (Heterogeneity of tumor microenvironment is associated with clinical prognosis of non-clear-cell renal cell carcinoma: a single-cell genomics study, CDDIS-21-2730RRR).

This following is the final author list and order, including additions and deletions. **Please reply whether agree or not as soon as possible.**

Heterogeneity of tumor microenvironment is associated with clinical prognosis of non-clear-cell renal cell carcinoma: a single-cell genomics study

Running title: scRNA presents nccRCC TME profile correlating with prognosis

Chen Wen-jin[1]†, Cao Hao[2,6†], Cao Jian-wei[3]†, Zuo Li[4]†, Qu Fa-jun[3], Xu Da[1], Zhang Hao[5], Gong Hai-yi[5], Chen Jia-xin[1], Ye Jian-qing[1], Gan Si-shun[1], Zhou Wang[1,3], Zhu Da-wei[4*], Pan Xiu-Wu[1,3]†, Cui Xin-gang

1. Department of Urology, The Third Affiliated Hospital of Second Military Medical University, 700 North Moyu Road, Shanghai 201805, China. chenwenjin@smmu.edu.cn (Chen Wen-jin); panxiuwu@126.co
(Xu Da); jiaxinchan@smmu.edu.cn (Chen Jia-xin); ye910@126.com (Ye Jian-qing); gansishun20101111@163.com (Gan Si-shun); brilliant212@163.com (Zhou Wang); cuixingang@smmu.edu.cn (Cui Xi
2. School of Life Science and Biopharmaceutics, Shenyang Pharmaceutical University, Shenyang 110016, Chinacaohao@syphu.edu.cn (Cao Hao)
3. Department of Urology, Xinhua Hospital, Shanghai Jiaotong University, School of Medicine, 1665 Kongjiang Road, Shanghai 200092, China. vit_c@126.com (Cao Jian-wei); 15900611435@126.com (Qu
4. Department of Urology, the Affiliated Changzhou No. 2 People's Hospital of Nanjing Medical University, 29 Xinglong Road, Changzhou, Jiangsu 213000, China. zuoli@njmu.edu.cn (Zuo Li); davysky@1
5. Department of Orthopedic Oncology, Changzheng Hospital of Second Military Medical University, 415 Fengyang Road, Shanghai 200003, China. doctor_zhanghao@163.com (Zhang Hao); geoff.ghy@12
6. Peking-Tsinghua Center for Life Sciences, TsinghuaUniversity, 100084 Beijing, China

† These authors contributed equally to this work and should be considered as co-first authors

*** Correspondence to:** Prof. Cui Xin-gang, M.D, Department of Urology, The Third Affiliated Hospital of Second Military Medical University, 700 North Moyu Road, Shanghai 201805, China; Department o
Jiaotong University, School of Medicine, 1665 Kongjiang Road, Shanghai 200092, China. Email address: cuixingang@smmu.edu.cn. Tel.: +86 02181887661; fax: +86 02181887661. Pan Xiu-Wu, M.D, Depar
Hospital of Second Military Medical University, 700 North Moyu Road, Shanghai 201805, China; Department of Urology, Xinhua Hospital, Shanghai Jiaotong University, School of Medicine, 1665 Kongjiang
address: panxiuwu@126.com. Zhu Da-wei, Department of Urology, the Affiliated Changzhou No. 2 People's Hospital of Nanjing Medical University, 29 Xinglong Road, Changzhou, Jiangsu 213000, China. En

Cui Xin-gang

主　题:	Re: Author list change	
发件人:	"左-立" <zuoli@njmu.edu.cn>	2021-12-24 20:29:00
收件人:	"崔心刚" <cuixingang@smmu.edu.cn>	

OK, I agree with the final author list and order, including additions and deletions

zuoli

邮箱：zuoli@njmu.edu.cn

签名由 网易邮箱大师 定制

On 12/24/2021 19:28, 崔心刚 wrote:

Colleagues and co-authors,

Our recent manuscript for ***Cell Death and Disease*** has been accepted (Heterogeneity of tumor microenvironment is associated with clinical prognosis of non-clear-cell renal cell carcinoma: a single-cell genomics study, CDDIS-21-2730RRR).

This following is the final author list and order, including additions and deletions. **Please reply whether agree or not as soon as possible.**

Cui Xin-gang

主　题:	Re: Author list change	
发件人:	"曲发军" <15900611435@126.com>	2021-12-24 20:25:32
收件人:	cuixingang <cuixingang@smmu.edu.cn>	

OK, I agree with the final author list and order, including additions and deletions。

---- 回复的原邮件 ----

发件人 崔心刚<cuixingang@smmu.edu.cn>

日期 2021年12月24日 19:28

收件人 chenwenjin@smmu.edu.cn<chenwenjin@smmu.edu.cn>、panxiuwu@126.com<panxiuwu@126.com>、vit_c@126.com<vit_c@126.com>、xuda@smmu.edu.cn<xuda@smmu.edu.cn>、jiaxinchan@smmu.edu.cn<jiaxinchan@smmu.edu.cn>、ye910@126.com<ye910@126.com>、gansishun20101111@163.com<gansishun20101111@163.com>、Chinacaohao@syphu.edu.cn<Chinacaohao@syphu.edu.cn>、15900611435@126.com<15900611435@126.com>、zuoli@njmu.edu.cn<zuoli@njmu.edu.cn>、davysky@163.com<davysky@163.com>、doctor_zhanghao@163.com<doctor_zhanghao@163.com>、geoff.ghy@126.com<geoff.ghy@126.com>、brilliant212@163.com<brilliant212@163.com>

主题 Author list change

Colleagues and co-authors,

Our recent manuscript for ***Cell Death and Disease*** has been accepted (Heterogeneity of tumor microenvironment is associated with clinical prognosis of non-clear-cell renal cell carcinoma: a single-cell genomics study, CDDIS-21-2730RRR).

This following is the final author list and order, including additions and deletions. **Please reply whether agree or not as soon as possible.**

Heterogeneity of tumor microenvironment is associated with clinical prognosis of non-clear-cell renal cell carcinoma: a single-cell genomics study

Running title: scRNA presents nccRCC TME profile correlating with prognosis

Chen Wen-jin[1]†, Cao Hao[2,6†], Cao Jian-wei[3]†, Zuo Li[4]†, Qu Fa-jun[3], Xu Da[1], Zhang Hao[5], Gong Hai-yi[5], Chen Jia-xin[1], Ye Jian-qing[1], Gan Si-shun[1], Zhou Wang[1,3], Zhu Da-wei[4*], Pan Xiu-Wu[1,3]†, Cui Xin-gang

1. Department of Urology, The Third Affiliated Hospital of Second Military Medical University, 700 North Moyu Road, Shanghai 201805, China. chenwenjin@smmu.edu.cn (Chen Wen-jin); panxiuwu@126.cc

(Xu Da); jiaxinchan@smmu.edu.cn (Chen Jia-xin); ye910@126.com (Ye Jian-qing); gansishun20101111@163.com (Gan Si-shun); brilliant212@163.com (Zhou Wang); cuixingang@smmu.edu.cn (Cui Xi

2. School of Life Science and Biopharmaceutics, Shenyang Pharmaceutical University, Shenyang 110016, Chinacaohao@syphu.edu.cn (Cao Hao)

3. Department of Urology, Xinhua Hospital, Shanghai Jiaotong University, School of Medicine, 1665 Kongjiang Road, Shanghai 200092, China. vit_c@126.com (Cao Jian-wei); 15900611435@126.com (Qu

4. Department of Urology, the Affiliated Changzhou No. 2 People's Hospital of Nanjing Medical University, 29 Xinglong Road, Changzhou, Jiangsu 213000, China. zuoli@njmu.edu.cn (Zuo Li); davysky@1

5. Department of Orthopedic Oncology, Changzheng Hospital of Second Military Medical University, 415 Fengyang Road, Shanghai 200003, China. doctor_zhanghao@163.com (Zhang Hao); geoff.ghy@12

6. Peking-Tsinghua Center for Life Sciences, TsinghuaUniversity, 100084 Beijing, China

† These authors contributed equally to this work and should be considered as co-first authors

*** Correspondence to:** Prof. Cui Xin-gang, M.D, Department of Urology, The Third Affiliated Hospital of Second Military Medical University, 700 North Moyu Road, Shanghai 201805, China; Department o

Jiaotong University, School of Medicine, 1665 Kongjiang Road, Shanghai 200092, China. Email address: cuixingang@smmu.edu.cn. Tel.: +86 02181887661; fax: +86 02181887661. Pan Xiu-Wu, M.D, Depar

Hospital of Second Military Medical University, 700 North Moyu Road, Shanghai 201805, China; Department of Urology, Xinhua Hospital, Shanghai Jiaotong University, School of Medicine, 1665 Kongjiang

address: panxiuwu@126.com. Zhu Da-wei, Department of Urology, the Affiliated Changzhou No. 2 People's Hospital of Nanjing Medical University, 29 Xinglong Road, Changzhou, Jiangsu 213000, China. En

Cui Xin-gang

主　题:	Re: Author list change	
发件人:	"徐达" <xuda@smmu.edu.cn>	2021-12-24 22:48:53
收件人:	"崔心刚" <cuixingang@smmu.edu.cn>	

OK, I agree with the final author list and order, including additions and deletions.

-----原始邮件-----
发件人:"崔心刚" <cuixingang@smmu.edu.cn>
发送时间:2021-12-24 19:28:06 (星期五)
收件人: chenwenjin@smmu.edu.cn, panxiuwu@126.com, vit_c@126.com, xuda@smmu.edu.cn, jiaxinchan@smmu.edu.cn, ye910@126.com, gansishun20101111@163.com, Chinacaohao@syphu.edu.cn, 15900611435@126.com, zuoli@njmu.edu.cn, davysky@163.com, doctor_zhanghao@163.com, geoff.ghy@126.com, brilliant212@163.com
抄送:
主题: Author list change

Colleagues and co-authors,

Our recent manuscript for ***Cell Death and Disease*** has been accepted (Heterogeneity of tumor microenvironment is associated with clinical prognosis of non-clear-cell renal cell carcinoma: a single-cell genomics study, CDDIS-21-2730RRR).

This following is the final author list and order, including additions and deletions. **Please reply whether agree or not as soon as possible.**

Heterogeneity of tumor microenvironment is associated with clinical prognosis of non-clear-cell renal cell carcinoma: a single-cell genomics study

Running title: scRNA presents nccRCC TME profile correlating with prognosis

Chen Wen-jin[1]†, Cao Hao[2,6†], Cao Jian-wei[3]†, Zuo Li[4]†, Qu Fa-jun[3], Xu Da[1], Zhang Hao[5], Gong Hai-yi[5], Chen Jia-xin[1], Ye Jian-qing[1], Gan Si-shun[1], Zhou Wang[1,3], Zhu Da-wei[4*], Pan Xiu-Wu[1,3]†, Cui Xin-gan

1. Department of Urology, The Third Affiliated Hospital of Second Military Medical University, 700 North Moyu Road, Shanghai 201805, China. chenwenjin@smmu.edu.cn (Chen Wen-jin); panxiuwu@126.c

(Xu Da); jiaxinchan@smmu.edu.cn (Chen Jia-xin); ye910@126.com (Ye Jian-qing); gansishun20101111@163.com (Gan Si-shun); brilliant212@163.com (Zhou Wang); cuixingang@smmu.edu.cn (Cui X

2. School of Life Science and Biopharmaceutics, Shenyang Pharmaceutical University, Shenyang 110016, Chinacaohao@syphu.edu.cn (Cao Hao)

3. Department of Urology, Xinhua Hospital, Shanghai Jiaotong University, School of Medicine, 1665 Kongjiang Road, Shanghai 200092, China. vit_c@126.com (Cao Jian-wei); 15900611435@126.com (Q

4. Department of Urology, the Affiliated Changzhou No. 2 People's Hospital of Nanjing Medical University, 29 Xinglong Road, Changzhou, Jiangsu 213000, China. zuoli@njmu.edu.cn (Zuo Li); davysky@

5. Department of Orthopedic Oncology, Changzheng Hospital of Second Military Medical University, 415 Fengyang Road, Shanghai 200003, China. doctor_zhanghao@163.com (Zhang Hao); geoff.ghy@1

6. Peking-Tsinghua Center for Life Sciences, TsinghuaUniversity, 100084 Beijing, China

† These authors contributed equally to this work and should be considered as co-first authors

*** Correspondence to:** Prof. Cui Xin-gang, M.D, Department of Urology, The Third Affiliated Hospital of Second Military Medical University, 700 North Moyu Road, Shanghai 201805, China; Department

Jiaotong University, School of Medicine, 1665 Kongjiang Road, Shanghai 200092, China. Email address: cuixingang@smmu.edu.cn. Tel.: +86 02181887661; fax: +86 02181887661. Pan Xiu-Wu, M.D, Depa

Hospital of Second Military Medical University, 700 North Moyu Road, Shanghai 201805, China; Department of Urology, Xinhua Hospital, Shanghai Jiaotong University, School of Medicine, 1665 Kongjian

address: panxiuwu@126.com. Zhu Da-wei, Department of Urology, the Affiliated Changzhou No. 2 People's Hospital of Nanjing Medical University, 29 Xinglong Road, Changzhou, Jiangsu 213000, China. E

Cui Xin-gang

主　题:	Re: Author list change	
发件人:	"Hao Zhang" <doctor_zhanghao@163.com>	2021-12-24 22:28:00
收件人:	"崔心刚" <cuixingang@smmu.edu.cn>	

OK, I agree with the final author list and order, including additions and deletions

---- Replied Message ----

From	崔心刚<cuixingang@smmu.edu.cn>
Date	12/24/2021 19:28
To	chenwenjin<chenwenjin@smmu.edu.cn>,<br>panxiuwu<panxiuwu@126.com>,<br>vit_c<vit_c@126.com>,<br>xuda<xuda@smmu.edu.cn>,<br>jiaxinchan<jiaxinchan@smmu.edu.cn>,<br>ye910<ye910@126.com>,<br>gansishun20101111<gansishun20101111@163.com>,<br>Chinacaohao<chinacaohao@syphu.edu.cn>,<br>15900611435<15900611435@126.com>,<br>zuoli<zuoli@njmu.edu.cn>,<br>davysky<davysky@163.com>,<br>doctor_zhanghao<doctor_zhanghao@163.com>,<br>geoff.ghy<geoff.ghy@126.com>,<br>brilliant212<brilliant212@163.com>
Subject	Author list change

Colleagues and co-authors,

Our recent manuscript for ***Cell Death and Disease*** has been accepted (Heterogeneity of tumor microenvironment is associated with clinical prognosis of non-clear-cell renal cell carcinoma: a single-cell genomics study, CDDIS-21-2730RRR).

This following is the final author list and order, including additions and deletions. **Please reply whether agree or not as soon as possible.**

Heterogeneity of tumor microenvironment is associated with clinical prognosis of non-clear-cell renal cell carcinoma: a single-cell genomics study

Running title: scRNA presents nccRCC TME profile correlating with prognosis

Chen Wen-jin[1]†, Cao Hao[2,6†], Cao Jian-wei[3]†, Zuo Li[4]†, Qu Fa-jun[3], Xu Da[1], Zhang Hao[5], Gong Hai-yi[5], Chen Jia-xin[1], Ye Jian-qing[1], Gan Si-shun[1], Zhou Wang[1,3], Zhu Da-wei[4*], Pan Xiu-Wu[1,3]†, Cui Xin-gang

1. Department of Urology, The Third Affiliated Hospital of Second Military Medical University, 700 North Moyu Road, Shanghai 201805, China. chenwenjin@smmu.edu.cn (Chen Wen-jin); panxiuwu@126.c[illegible]
(Xu Da); jiaxinchan@smmu.edu.cn (Chen Jia-xin); ye910@126.com (Ye Jian-qing); gansishun20101111@163.com (Gan Si-shun); brilliant212@163.com (Zhou Wang); cuixingang@smmu.edu.cn (Cui Xi[illegible]
2. School of Life Science and Biopharmaceutics, Shenyang Pharmaceutical University, Shenyang 110016, Chinacaohao@syphu.edu.cn (Cao Hao)
3. Department of Urology, Xinhua Hospital, Shanghai Jiaotong University, School of Medicine, 1665 Kongjiang Road, Shanghai 200092, China. vit_c@126.com (Cao Jian-wei); 15900611435@126.com (Qu[illegible]
4. Department of Urology, the Affiliated Changzhou No. 2 People's Hospital of Nanjing Medical University, 29 Xinglong Road, Changzhou, Jiangsu 213000, China. zuoli@njmu.edu.cn (Zuo Li); davysky@1[illegible]
5. Department of Orthopedic Oncology, Changzheng Hospital of Second Military Medical University, 415 Fengyang Road, Shanghai 200003, China. doctor_zhanghao@163.com (Zhang Hao); geoff.ghy@12[illegible]
6. Peking-Tsinghua Center for Life Sciences, TsinghuaUniversity, 100084 Beijing, China

† These authors contributed equally to this work and should be considered as co-first authors

*** Correspondence to:** Prof. Cui Xin-gang, M.D, Department of Urology, The Third Affiliated Hospital of Second Military Medical University, 700 North Moyu Road, Shanghai 201805, China; Department [illegible]
Jiaotong University, School of Medicine, 1665 Kongjiang Road, Shanghai 200092, China. Email address: cuixingang@smmu.edu.cn. Tel.: +86 02181887661; fax: +86 02181887661. Pan Xiu-Wu, M.D, Depar[illegible]
Hospital of Second Military Medical University, 700 North Moyu Road, Shanghai 201805, China; Department of Urology, Xinhua Hospital, Shanghai Jiaotong University, School of Medicine, 1665 Kongjiang [illegible]
address: panxiuwu@126.com. Zhu Da-wei, Department of Urology, the Affiliated Changzhou No. 2 People's Hospital of Nanjing Medical University, 29 Xinglong Road, Changzhou, Jiangsu 213000, China. Em[illegible]

主　题:	Re: Author list change
发件人:	"Haiyi Gong" <geoff.ghy@126.com> 2021-12-24 21:45:11
收件人:	gansishun20101111 <gansishun20101111@163.com>
抄　送:	cuixingang <cuixingang@smmu.edu.cn>, chenwenjin <chenwenjin@smmu.edu.cn>, panxiuwu <panxiuwu@126.com>, vit_c <vit_c@126.com>, xuda <xuda@smmu.edu.cn>, jiaxinchan <jiaxinchan@smmu.edu.cn>, ye910 <ye910@126.com>, Chinacaohao <Chinacaohao@syphu.edu.cn>, 15900611435 <15900611435@126.com>, zuoli <zuoli@njmu.edu.cn>, davysky <davysky@163.com>, doctor_zhanghao <doctor_zhanghao@163.com>, brilliant212 <brilliant212@163.com>

OK, I agree with the final author list and order, including additions and deletions.

---- 回复的原邮件 ----

发件人	干思舜<gansishun20101111@163.com>
日期	2021年12月24日 21:12
收件人	崔心刚<cuixingang@smmu.edu.cn>
抄送至	chenwenjin@smmu.edu.cn<chenwenjin@smmu.edu.cn>、panxiuwu@126.com<panxiuwu@126.com>、vit_c@126.com<vit_c@126.com>、xuda@smmu.edu.cn<xuda@smmu.edu.cn>、jiaxinchan@smmu.edu.cn<jiaxinchan@smmu.edu.cn>、ye910@126.com<ye910@126.com>、Chinacaohao@syphu.edu.cn<Chinacaohao@syphu.edu.cn>、15900611435@126.com<15900611435@126.com>、zuoli@njmu.edu.cn<zuoli@njmu.edu.cn>、davysky@163.com<davysky@163.com>、doctor_zhanghao@163.com<doctor_zhanghao@163.com>、geoff.ghy@126.com<geoff.ghy@126.com>、brilliant212@163.com<brilliant212@163.com>
主题	Re:Author list change

OK,I agree with the final author list and order,including additions and deletions.

At 2021-12-24 19:28:06, "崔心刚" <cuixingang@smmu.edu.cn> wrote:

Colleagues and co-authors,

Our recent manuscript for ***Cell Death and Disease*** has been accepted (Heterogeneity of tumor microenvironment is associated with clinical prognosis of non-clear-cell renal cell carcinoma: a single-cell genomics study, CDDIS-21-2730RRR).

This following is the final author list and order, including additions and deletions. **Please reply whether agree or not as soon as possible.**

Heterogeneity of tumor microenvironment is associated with clinical prognosis of non-clear-cell renal cell carcinoma: a single-cell genomics study

Running title: scRNA presents nccRCC TME profile correlating with prognosis

Chen Wen-jin[1]†, Cao Hao[2,6†], Cao Jian-wei[3]†, Zuo Li[4]†, Qu Fa-jun[3], Xu Da[1], Zhang Hao[5], Gong Hai-yi[5], Chen Jia-xin[1], Ye Jian-qing[1], Gan Si-shun[1], Zhou Wang[1,3], Zhu Da-wei[4*], Pan Xiu-Wu[1,3]†, Cui Xin-gan

1. Department of Urology, The Third Affiliated Hospital of Second Military Medical University, 700 North Moyu Road, Shanghai 201805, China. chenwenjin@smmu.edu.cn (Chen Wen-jin); panxiuwu@126.c
   (Xu Da); jiaxinchan@smmu.edu.cn (Chen Jia-xin); ye910@126.com (Ye Jian-qing); gansishun20101111@163.com (Gan Si-shun); brilliant212@163.com (Zhou Wang); cuixingang@smmu.edu.cn (Cui X
2. School of Life Science and Biopharmaceutics, Shenyang Pharmaceutical University, Shenyang 110016, Chinacaohao@syphu.edu.cn (Cao Hao)
3. Department of Urology, Xinhua Hospital, Shanghai Jiaotong University, School of Medicine, 1665 Kongjiang Road, Shanghai 200092, China. vit_c@126.com (Cao Jian-wei); 15900611435@126.com (Q
4. Department of Urology, the Affiliated Changzhou No. 2 People's Hospital of Nanjing Medical University, 29 Xinglong Road, Changzhou, Jiangsu 213000, China. zuoli@njmu.edu.cn (Zuo Li); davysky@
5. Department of Orthopedic Oncology, Changzheng Hospital of Second Military Medical University, 415 Fengyang Road, Shanghai 200003, China. doctor_zhanghao@163.com (Zhang Hao); geoff.ghy@1
6. Peking-Tsinghua Center for Life Sciences, TsinghuaUniversity, 100084 Beijing, China

† These authors contributed equally to this work and should be considered as co-first authors

*** Correspondence to:** Prof. Cui Xin-gang, M.D, Department of Urology, The Third Affiliated Hospital of Second Military Medical University, 700 North Moyu Road, Shanghai 201805, China; Department
Jiaotong University, School of Medicine, 1665 Kongjiang Road, Shanghai 200092, China. Email address: cuixingang@smmu.edu.cn. Tel.: +86 02181887661; fax: +86 02181887661. Pan Xiu-Wu, M.D, Depa
Hospital of Second Military Medical University, 700 North Moyu Road, Shanghai 201805, China; Department of Urology, Xinhua Hospital, Shanghai Jiaotong University, School of Medicine, 1665 Kongjian
address: panxiuwu@126.com. Zhu Da-wei, Department of Urology, the Affiliated Changzhou No. 2 People's Hospital of Nanjing Medical University, 29 Xinglong Road, Changzhou, Jiangsu 213000, China. E

Cui Xin-gang

主　题:	Re: Author list change
发件人:	"陈佳鑫" <jiaxinchan@smmu.edu.cn> 2021-12-24 22:07:32
收件人:	"崔心刚" <cuixingang@smmu.edu.cn>

OK, I agree with the final author list and order, including additions and deletions.

-----原始邮件-----
发件人:"崔心刚" <cuixingang@smmu.edu.cn>
发送时间:2021-12-24 19:28:06 (星期五)
收件人: chenwenjin@smmu.edu.cn, panxiuwu@126.com, vit_c@126.com, xuda@smmu.edu.cn, jiaxinchan@smmu.edu.cn, ye910@126.com, gansishun20101111@163.com, Chinacaohao@syphu.edu.cn, 15900611435@126.com, zuoli@njmu.edu.cn, davysky@163.com, doctor_zhanghao@163.com, geoff.ghy@126.com, brilliant212@163.com
抄送:
主题: Author list change

Colleagues and co-authors,

Our recent manuscript for ***Cell Death and Disease*** has been accepted (Heterogeneity of tumor microenvironment is associated with clinical prognosis of non-clear-cell renal cell carcinoma: a single-cell genomics study, CDDIS-21-2730RRR).

This following is the final author list and order, including additions and deletions. **Please reply whether agree or not as soon as possible.**

Heterogeneity of tumor microenvironment is associated with clinical prognosis of non-clear-cell renal cell carcinoma: a single-cell genomics study

Running title: scRNA presents nccRCC TME profile correlating with prognosis

Chen Wen-jin[1]†, Cao Hao[2,6†], Cao Jian-wei[3]†, Zuo Li[4]†, Qu Fa-jun[3], Xu Da[1], Zhang Hao[5], Gong Hai-yi[5], Chen Jia-xin[1], Ye Jian-qing[1], Gan Si-shun[1], Zhou Wang[1,3], Zhu Da-wei[4*], Pan Xiu-Wu[1,3]†, Cui Xin-gan

1. Department of Urology, The Third Affiliated Hospital of Second Military Medical University, 700 North Moyu Road, Shanghai 201805, China. chenwenjin@smmu.edu.cn (Chen Wen-jin); panxiuwu@126.c

(Xu Da); jiaxinchan@smmu.edu.cn (Chen Jia-xin); ye910@126.com (Ye Jian-qing); gansishun20101111@163.com (Gan Si-shun); brilliant212@163.com (Zhou Wang); cuixingang@smmu.edu.cn (Cui X

2. School of Life Science and Biopharmaceutics, Shenyang Pharmaceutical University, Shenyang 110016, Chinacaohao@syphu.edu.cn (Cao Hao)

3. Department of Urology, Xinhua Hospital, Shanghai Jiaotong University, School of Medicine, 1665 Kongjiang Road, Shanghai 200092, China. vit_c@126.com (Cao Jian-wei); 15900611435@126.com (Q

4. Department of Urology, the Affiliated Changzhou No. 2 People's Hospital of Nanjing Medical University, 29 Xinglong Road, Changzhou, Jiangsu 213000, China. zuoli@njmu.edu.cn (Zuo Li); davysky@

5. Department of Orthopedic Oncology, Changzheng Hospital of Second Military Medical University, 415 Fengyang Road, Shanghai 200003, China. doctor_zhanghao@163.com (Zhang Hao); geoff.ghy@1

6. Peking-Tsinghua Center for Life Sciences, TsinghuaUniversity, 100084 Beijing, China

† These authors contributed equally to this work and should be considered as co-first authors

*** Correspondence to:** Prof. Cui Xin-gang, M.D, Department of Urology, The Third Affiliated Hospital of Second Military Medical University, 700 North Moyu Road, Shanghai 201805, China; Department

Jiaotong University, School of Medicine, 1665 Kongjiang Road, Shanghai 200092, China. Email address: cuixingang@smmu.edu.cn. Tel.: +86 02181887661; fax: +86 02181887661. Pan Xiu-Wu, M.D, Depa

Hospital of Second Military Medical University, 700 North Moyu Road, Shanghai 201805, China; Department of Urology, Xinhua Hospital, Shanghai Jiaotong University, School of Medicine, 1665 Kongjian

address: panxiuwu@126.com. Zhu Da-wei, Department of Urology, the Affiliated Changzhou No. 2 People's Hospital of Nanjing Medical University, 29 Xinglong Road, Changzhou, Jiangsu 213000, China. E

Cui Xin-gang

主　题:	Re:Author list change	
发件人:	"叶" <ye910@126.com>	2021-12-24 21:00:29
收件人:	"崔心刚" <cuixingang@smmu.edu.cn>	

OK, I agree with the final author list and order, including additions and deletions.

At 2021-12-24 19:28:06, "崔心刚" <cuixingang@smmu.edu.cn> wrote:

> Colleagues and co-authors,
>
> Our recent manuscript for ***Cell Death and Disease*** has been accepted (Heterogeneity of tumor microenvironment is associated with clinical prognosis of non-clear-cell renal cell carcinoma: a single-cell genomics study, CDDIS-21-2730RRR).
>
> This following is the final author list and order, including additions and deletions. **Please reply whether agree or not as soon as possible.**

Heterogeneity of tumor microenvironment is associated with clinical prognosis of non-clear-cell renal cell carcinoma: a single-cell genomics study

Running title: scRNA presents nccRCC TME profile correlating with prognosis

Chen Wen-jin[1]†, Cao Hao[2,6]†, Cao Jian-wei[3]†, Zuo Li[4]†, Qu Fa-jun[3], Xu Da[1], Zhang Hao[5], Gong Hai-yi[5], Chen Jia-xin[1], Ye Jian-qing[1], Gan Si-shun[1], Zhou Wang[1,3], Zhu Da-wei[4*], Pan Xiu-Wu[1,3]†, Cui Xin-gan

1. Department of Urology, The Third Affiliated Hospital of Second Military Medical University, 700 North Moyu Road, Shanghai 201805, China. chenwenjin@smmu.edu.cn (Chen Wen-jin); panxiuwu@126.

   (Xu Da); jiaxinchan@smmu.edu.cn (Chen Jia-xin); ye910@126.com (Ye Jian-qing); gansishun20101111@163.com (Gan Si-shun); brilliant212@163.com (Zhou Wang); cuixingang@smmu.edu.cn (Cui X

2. School of Life Science and Biopharmaceutics, Shenyang Pharmaceutical University, Shenyang 110016, Chinacaohao@syphu.edu.cn (Cao Hao)

3. Department of Urology, Xinhua Hospital, Shanghai Jiaotong University, School of Medicine, 1665 Kongjiang Road, Shanghai 200092, China. vit_c@126.com (Cao Jian-wei); 15900611435@126.com (Q

4. Department of Urology, the Affiliated Changzhou No. 2 People's Hospital of Nanjing Medical University, 29 Xinglong Road, Changzhou, Jiangsu 213000, China. zuoli@njmu.edu.cn (Zuo Li); davysky@

5. Department of Orthopedic Oncology, Changzheng Hospital of Second Military Medical University, 415 Fengyang Road, Shanghai 200003, China. doctor_zhanghao@163.com (Zhang Hao); geoff.ghy@1

6. Peking-Tsinghua Center for Life Sciences, TsinghuaUniversity, 100084 Beijing, China

† These authors contributed equally to this work and should be considered as co-first authors

*** Correspondence to:** Prof. Cui Xin-gang, M.D, Department of Urology, The Third Affiliated Hospital of Second Military Medical University, 700 North Moyu Road, Shanghai 201805, China; Department

Jiaotong University, School of Medicine, 1665 Kongjiang Road, Shanghai 200092, China. Email address: cuixingang@smmu.edu.cn. Tel.: +86 02181887661; fax: +86 02181887661. Pan Xiu-Wu, M.D, Depa

Hospital of Second Military Medical University, 700 North Moyu Road, Shanghai 201805, China; Department of Urology, Xinhua Hospital, Shanghai Jiaotong University, School of Medicine, 1665 Kongjian

address: panxiuwu@126.com. Zhu Da-wei, Department of Urology, the Affiliated Changzhou No. 2 People's Hospital of Nanjing Medical University, 29 Xinglong Road, Changzhou, Jiangsu 213000, China. E

Cui Xin-gang

主　题:	Re:Author list change
发件人:	"干思舜" <gansishun20101111@163.com> 2021-12-24 21:12:11
收件人:	"崔心刚" <cuixingang@smmu.edu.cn>
抄　送:	chenwenjin@smmu.edu.cn, panxiuwu@126.com, vit_c@126.com, xuda@smmu.edu.cn, jiaxinchan@smmu.edu.cn, ye910@126.com, Chinacaohao@syphu.edu.cn, 15900611435@126.com, zuoli@njmu.edu.cn, davysky@163.com, doctor_zhanghao@163.com, geoff.ghy@126.com, brilliant212@163.com

OK,I agree with the final author list and order,including additions and deletions.

At 2021-12-24 19:28:06, "崔心刚" <cuixingang@smmu.edu.cn> wrote:

> Colleagues and co-authors,
>
> Our recent manuscript for ***Cell Death and Disease*** has been accepted (Heterogeneity of tumor microenvironment is associated with clinical prognosis of non-clear-cell renal cell carcinoma: a single-cell genomics study, CDDIS-21-2730RRR).
>
> This following is the final author list and order, including additions and deletions. **Please reply whether agree or not as soon as possible.**

Heterogeneity of tumor microenvironment is associated with clinical prognosis of non-clear-cell renal cell carcinoma: a single-cell genomics study

Running title: scRNA presents nccRCC TME profile correlating with prognosis

Chen Wen-jin[1]†, Cao Hao[2,6†], Cao Jian-wei[3]†, Zuo Li[4]†, Qu Fa-jun[3], Xu Da[1], Zhang Hao[5], Gong Hai-yi[5], Chen Jia-xin[1], Ye Jian-qing[1], Gan Si-shun[1], Zhou Wang[1,3], Zhu Da-wei[4*], Pan Xiu-Wu[1,3]†, Cui Xin-gan

1. Department of Urology, The Third Affiliated Hospital of Second Military Medical University, 700 North Moyu Road, Shanghai 201805, China. chenwenjin@smmu.edu.cn (Chen Wen-jin); panxiuwu@126.c

   (Xu Da); jiaxinchan@smmu.edu.cn (Chen Jia-xin); ye910@126.com (Ye Jian-qing); gansishun20101111@163.com (Gan Si-shun); brilliant212@163.com (Zhou Wang); cuixingang@smmu.edu.cn (Cui X

2. School of Life Science and Biopharmaceutics, Shenyang Pharmaceutical University, Shenyang 110016, Chinacaohao@syphu.edu.cn (Cao Hao)

3. Department of Urology, Xinhua Hospital, Shanghai Jiaotong University, School of Medicine, 1665 Kongjiang Road, Shanghai 200092, China. yjt_c@126.com (Cao Jian-wei); 15900611435@126.com (Q

4. Department of Urology, the Affiliated Changzhou No. 2 People's Hospital of Nanjing Medical University, 29 Xinglong Road, Changzhou, Jiangsu 213000, China. zuoli@njmu.edu.cn (Zuo Li); davysky@

5. Department of Orthopedic Oncology, Changzheng Hospital of Second Military Medical University, 415 Fengyang Road, Shanghai 200003, China. doctor_zhanghao@163.com (Zhang Hao); geoff.ghy@1

6. Peking-Tsinghua Center for Life Sciences, TsinghuaUniversity, 100084 Beijing, China

† These authors contributed equally to this work and should be considered as co-first authors

*** Correspondence to:** Prof. Cui Xin-gang, M.D, Department of Urology, The Third Affiliated Hospital of Second Military Medical University, 700 North Moyu Road, Shanghai 201805, China; Department

Jiaotong University, School of Medicine, 1665 Kongjiang Road, Shanghai 200092, China. Email address: cuixingang@smmu.edu.cn. Tel.: +86 02181887661; fax: +86 02181887661. Pan Xiu-Wu, M.D, Depa

Hospital of Second Military Medical University, 700 North Moyu Road, Shanghai 201805, China; Department of Urology, Xinhua Hospital, Shanghai Jiaotong University, School of Medicine, 1665 Kongjian

address: panxiuwu@126.com. Zhu Da-wei, Department of Urology, the Affiliated Changzhou No. 2 People's Hospital of Nanjing Medical University, 29 Xinglong Road, Changzhou, Jiangsu 213000, China. E

Cui Xin-gang

主　题:	Re: Author list change	
发件人:	wang <brilliant212@163.com>	2021-12-24 20:11:35
收件人:	cuixingang <cuixingang@smmu.edu.cn>	

OK, I agree with the final author list and order, including additions and deletions.

---- 回复的原邮件 ----

发件人	崔心刚<cuixingang@smmu.edu.cn>
日期	2021年12月24日 19:28
收件人	chenwenjin@smmu.edu.cn<chenwenjin@smmu.edu.cn>、panxiuwu@126.com<panxiuwu@126.com>、vit_c@126.com<vit_c@126.com>、xuda@smmu.edu.cn<xuda@smmu.edu.cn>、jiaxinchan@smmu.edu.cn<jiaxinchan@smmu.edu.cn>、ye910@126.com<ye910@126.com>、gansishun20101111@163.com<gansishun20101111@163.com>、Chinacaohao@syphu.edu.cn<Chinacaohao@syphu.edu.cn>、15900611435@126.com<15900611435@126.com>、zuoli@njmu.edu.cn<zuoli@njmu.edu.cn>、davysky@163.com<davysky@163.com>、doctor_zhanghao@163.com<doctor_zhanghao@163.com>、geoff.ghy@126.com<geoff.ghy@126.com>、brilliant212@163.com<brilliant212@163.com>
主题	Author list change

Colleagues and co-authors,

Our recent manuscript for ***Cell Death and Disease*** has been accepted (Heterogeneity of tumor microenvironment is associated with clinical prognosis of non-clear-cell renal cell carcinoma: a single-cell genomics study, CDDIS-21-2730RRR).

This following is the final author list and order, including additions and deletions. **Please reply whether agree or not as soon as possible.**

Heterogeneity of tumor microenvironment is associated with clinical prognosis of non-clear-cell renal cell carcinoma: a single-cell genomics study

Running title: scRNA presents nccRCC TME profile correlating with prognosis

Chen Wen-jin[1]†, Cao Hao[2,6†], Cao Jian-wei[3]†, Zuo Li[4]†, Qu Fa-jun[3], Xu Da[1], Zhang Hao[5], Gong Hai-yi[5], Chen Jia-xin[1], Ye Jian-qing[1], Gan Si-shun[1], Zhou Wang[1,3], Zhu Da-wei[4*], Pan Xiu-Wu[1,3]†, Cui Xin-gang

1. Department of Urology, The Third Affiliated Hospital of Second Military Medical University, 700 North Moyu Road, Shanghai 201805, China. chenwenjin@smmu.edu.cn (Chen Wen-jin); panxiuwu@126.co
(Xu Da); jiaxinchan@smmu.edu.cn (Chen Jia-xin); ye910@126.com (Ye Jian-qing); gansishun20101111@163.com (Gan Si-shun); brilliant212@163.com (Zhou Wang); cuixingang@smmu.edu.cn (Cui Xi
2. School of Life Science and Biopharmaceutics, Shenyang Pharmaceutical University, Shenyang 110016, Chinacaohao@syphu.edu.cn (Cao Hao)
3. Department of Urology, Xinhua Hospital, Shanghai Jiaotong University, School of Medicine, 1665 Kongjiang Road, Shanghai 200092, China. vit_c@126.com (Cao Jian-wei); 15900611435@126.com (Qu
4. Department of Urology, the Affiliated Changzhou No. 2 People's Hospital of Nanjing Medical University, 29 Xinglong Road, Changzhou, Jiangsu 213000, China. zuoli@njmu.edu.cn (Zuo Li); davysky@1
5. Department of Orthopedic Oncology, Changzheng Hospital of Second Military Medical University, 415 Fengyang Road, Shanghai 200003, China. doctor_zhanghao@163.com (Zhang Hao); geoff.ghy@12
6. Peking-Tsinghua Center for Life Sciences, TsinghuaUniversity, 100084 Beijing, China

† These authors contributed equally to this work and should be considered as co-first authors

*** Correspondence to:** Prof. Cui Xin-gang, M.D, Department of Urology, The Third Affiliated Hospital of Second Military Medical University, 700 North Moyu Road, Shanghai 201805, China; Department o
Jiaotong University, School of Medicine, 1665 Kongjiang Road, Shanghai 200092, China. Email address: cuixingang@smmu.edu.cn. Tel.: +86 02181887661; fax: +86 02181887661. Pan Xiu-Wu, M.D, Depar
Hospital of Second Military Medical University, 700 North Moyu Road, Shanghai 201805, China; Department of Urology, Xinhua Hospital, Shanghai Jiaotong University, School of Medicine, 1665 Kongjiang
address: panxiuwu@126.com. Zhu Da-wei, Department of Urology, the Affiliated Changzhou No. 2 People's Hospital of Nanjing Medical University, 29 Xinglong Road, Changzhou, Jiangsu 213000, China. En

Cui Xin-gang

主　题:	Re:Author list change	
发件人:	"潘秀武" <panxiuwu@126.com>	2021-12-24 20:07:10
收件人:	"崔心刚" <cuixingang@smmu.edu.cn>	

OK, I agree with the final author list and order, including additions and deletions

At 2021-12-24 19:28:06, "崔心刚" <cuixingang@smmu.edu.cn> wrote:

> Colleagues and co-authors,
>
> Our recent manuscript for ***Cell Death and Disease*** has been accepted (Heterogeneity of tumor microenvironment is associated with clinical prognosis of non-clear-cell renal cell carcinoma: a single-cell genomics study, CDDIS-21-2730RRR).
>
> This following is the final author list and order, including additions and deletions. **Please reply whether agree or not as soon as possible.**

Heterogeneity of tumor microenvironment is associated with clinical prognosis of non-clear-cell renal cell carcinoma: a single-cell genomics study

Running title: scRNA presents nccRCC TME profile correlating with prognosis

Chen Wen-jin[1]†, Cao Hao[2,6†], Cao Jian-wei[3]†, Zuo Li[4]†, Qu Fa-jun[3], Xu Da[1], Zhang Hao[5], Gong Hai-yi[5], Chen Jia-xin[1], Ye Jian-qing[1], Gan Si-shun[1], Zhou Wang[1,3], Zhu Da-wei[4*], Pan Xiu-Wu[1,3]†, Cui Xin-gan

1. Department of Urology, The Third Affiliated Hospital of Second Military Medical University, 700 North Moyu Road, Shanghai 201805, China. chenwenjin@smmu.edu.cn (Chen Wen-jin); panxiuwu@126.

   (Xu Da); jiaxinchan@smmu.edu.cn (Chen Jia-xin); ye910@126.com (Ye Jian-qing); gansishun20101111@163.com (Gan Si-shun); brilliant212@163.com (Zhou Wang); cuixingang@smmu.edu.cn (Cui X
2. School of Life Science and Biopharmaceutics, Shenyang Pharmaceutical University, Shenyang 110016, Chinacaohao@syphu.edu.cn (Cao Hao)
3. Department of Urology, Xinhua Hospital, Shanghai Jiaotong University, School of Medicine, 1665 Kongjiang Road, Shanghai 200092, China. vit_c@126.com (Cao Jian-wei); 15900611435@126.com (Q
4. Department of Urology, the Affiliated Changzhou No. 2 People's Hospital of Nanjing Medical University, 29 Xinglong Road, Changzhou, Jiangsu 213000, China. zuoli@njmu.edu.cn (Zuo Li); davysky@
5. Department of Orthopedic Oncology, Changzheng Hospital of Second Military Medical University, 415 Fengyang Road, Shanghai 200003, China. doctor_zhanghao@163.com (Zhang Hao); geoff.ghy@1
6. Peking-Tsinghua Center for Life Sciences, TsinghuaUniversity, 100084 Beijing, China

† These authors contributed equally to this work and should be considered as co-first authors

* **Correspondence to:** Prof. Cui Xin-gang, M.D, Department of Urology, The Third Affiliated Hospital of Second Military Medical University, 700 North Moyu Road, Shanghai 201805, China; Department

Jiaotong University, School of Medicine, 1665 Kongjiang Road, Shanghai 200092, China. Email address: cuixingang@smmu.edu.cn. Tel.: +86 02181887661; fax: +86 02181887661. Pan Xiu-Wu, M.D, Depa

Hospital of Second Military Medical University, 700 North Moyu Road, Shanghai 201805, China; Department of Urology, Xinhua Hospital, Shanghai Jiaotong University, School of Medicine, 1665 Kongjian

address: panxiuwu@126.com. Zhu Da-wei, Department of Urology, the Affiliated Changzhou No. 2 People's Hospital of Nanjing Medical University, 29 Xinglong Road, Changzhou, Jiangsu 213000, China. E

Cui Xin-gang

主　题:	Re: Author list change	
发件人:	davysky <davysky@163.com>	2021-12-25 10:03:51
收件人:	"崔心刚" <cuixingang@smmu.edu.cn>	

OK, I agree with the final author list and order, including additions and deletions

On 12/24/2021 19:28, 崔心刚 wrote:

Colleagues and co-authors,

Our recent manuscript for ***Cell Death and Disease*** has been accepted (Heterogeneity of tumor microenvironment is associated with clinical prognosis of non-clear-cell renal cell carcinoma: a single-cell genomics study, CDDIS-21-2730RRR).

This following is the final author list and order, including additions and deletions. **Please reply whether agree or not as soon as possible.**

Heterogeneity of tumor microenvironment is associated with clinical prognosis of non-clear-cell renal cell carcinoma: a single-cell genomics study

Running title: scRNA presents nccRCC TME profile correlating with prognosis

Chen Wen-jin[1]†, Cao Hao[2,6†], Cao Jian-wei[3]†, Zuo Li[4]†, Qu Fa-jun[3], Xu Da[1], Zhang Hao[5], Gong Hai-yi[5], Chen Jia-xin[1], Ye Jian-qing[1], Gan Si-shun[1], Zhou Wang[1,3], Zhu Da-wei[4*], Pan Xiu-Wu[1,3]†, Cui Xin-gang

1. Department of Urology, The Third Affiliated Hospital of Second Military Medical University, 700 North Moyu Road, Shanghai 201805, China. chenwenjin@smmu.edu.cn (Chen Wen-jin); panxiuwu@126.c

   (Xu Da); jiaxinchan@smmu.edu.cn (Chen Jia-xin); ye910@126.com (Ye Jian-qing); gansishun20101111@163.com (Gan Si-shun); brilliant212@163.com (Zhou Wang); cuixingang@smmu.edu.cn (Cui Xi

2. School of Life Science and Biopharmaceutics, Shenyang Pharmaceutical University, Shenyang 110016, Chinacaohao@syphu.edu.cn (Cao Hao)

3. Department of Urology, Xinhua Hospital, Shanghai Jiaotong University, School of Medicine, 1665 Kongjiang Road, Shanghai 200092, China. vit_c@126.com (Cao Jian-wei); 15900611435@126.com (Qu

4. Department of Urology, the Affiliated Changzhou No. 2 People's Hospital of Nanjing Medical University, 29 Xinglong Road, Changzhou, Jiangsu 213000, China. zuoli@njmu.edu.cn (Zuo Li); davysky@1

5. Department of Orthopedic Oncology, Changzheng Hospital of Second Military Medical University, 415 Fengyang Road, Shanghai 200003, China. doctor_zhanghao@163.com (Zhang Hao); geoff.ghy@12

6. Peking-Tsinghua Center for Life Sciences, TsinghuaUniversity, 100084 Beijing, China

† These authors contributed equally to this work and should be considered as co-first authors

*** Correspondence to:** Prof. Cui Xin-gang, M.D, Department of Urology, The Third Affiliated Hospital of Second Military Medical University, 700 North Moyu Road, Shanghai 201805, China; Department

Jiaotong University, School of Medicine, 1665 Kongjiang Road, Shanghai 200092, China. Email address: cuixingang@smmu.edu.cn. Tel.: +86 02181887661; fax: +86 02181887661. Pan Xiu-Wu, M.D, Depar

Hospital of Second Military Medical University, 700 North Moyu Road, Shanghai 201805, China; Department of Urology, Xinhua Hospital, Shanghai Jiaotong University, School of Medicine, 1665 Kongjiang

address: panxiuwu@126.com. Zhu Da-wei, Department of Urology, the Affiliated Changzhou No. 2 People's Hospital of Nanjing Medical University, 29 Xinglong Road, Changzhou, Jiangsu 213000, China. En

Cui Xin-gang
